# Whole Exome Sequencing of Multi-Regional Biopsies from Metastatic Lesions to Evaluate Actionable Truncal Mutations Using a Single-Pass Percutaneous Technique

**DOI:** 10.3390/cancers12061599

**Published:** 2020-06-17

**Authors:** Valerie Heong, Darwin Tay, Shane Ee Goh, Bernard Wee, Tuan Zea Tan, Ross Soo, Brendan Pang, Diana Lim, Anil Gopinathan, Samuel Ow, Cheng Ean Chee, Boon Cher Goh, Soo Chin Lee, Wei Peng Yong, Andrea Wong, Mohamed Feroz Mohd Omar, Richie Soong, David S.P. Tan

**Affiliations:** 1 Department of Haematology-Oncology, National University Cancer Institute, Singapore 119074, Singapore; valerie_ym_heong@ttsh.com.sg (V.H.); ross_soo@nuhs.edu.sg (R.S.); samuel_ow@nuhs.edu.sg (S.O.); cheng_ean_chee@nuhs.edu.sg (C.E.C.); phcgbc@nus.edu.sg (B.C.G.); soo_chin_lee@nuhs.edu.sg (S.C.L.); Wei_Peng_YONG@nuhs.edu.sg (W.P.Y.); Andrea_LA_WONG@nuhs.edu.sg (A.W.); 2 Cancer Science Institute of Singapore, National University of Singapore, Singapore 117599, Singapore; dwintay@gmail.com (D.T.); shanegoh8@gmail.com (S.E.G.); csittz@nus.edu.sg (T.Z.T.); feroz.omar@u.nus.edu (M.F.M.O.); richiesoong@gmail.com (R.S.); 3 Department of Radiology, National University Hospital, Singapore 119074, Singapore; bernard_bk_wee@nuhs.edu.sg (B.W.); anil_gopinathan@nuhs.edu.sg (A.G.); 4 Department of Pathology, National University of Singapore, Singapore 119077, Singapore; brendan.pang@parkwaypantai.com (B.P.); diana_gz_lim@nuhs.edu.sg (D.L.)

**Keywords:** intratumor heterogeneity, multiple biopsies, tumor evolution, clonality classification, strategic therapeutic intervention

## Abstract

We investigate the feasibility of obtaining multiple spatially-separated biopsies from a single lesion to explore intratumor heterogeneity and identify actionable truncal mutations using whole exome sequencing (WES). A single-pass radiologically-guided percutaneous technique was used to obtain four spatially-separated biopsies from a single metastatic lesion. WES was performed to identify putative truncal variants (PTVs), defined as a non-synonymous somatic (NSS) variant present in all four spatially separated biopsies. Actionable truncal mutations—filtered using the FoundationOne panel—were defined as clinically relevant PTVs. Mutational landscapes of each biopsy and their association with patient outcomes were assessed. WES on 50 biopsied samples from 13 patients across six cancer types were analyzed. Actionable truncal mutations were identified in 9/13 patients; 31.1 ± 5.12 more unique NSS variants were detected with every additional multi- region tumor biopsy (MRTB) analyzed. The number of PTVs dropped by 16.1 ± 17.9 with every additional MRTB, with the decrease most pronounced (36.8 ± 19.7) when two MRTB were analyzed compared to one. MRTB most reliably predicted PTV compared to in silico analysis of allele frequencies and cancer cell fraction based on one biopsy sample. Three patients treated with actionable truncal mutation-directed therapy derived clinical benefit. Multi-regional sampling for genomics analysis is feasible and informative to help prioritize precision-therapy strategies.

## 1. Introduction

Intratumor heterogeneity is a key challenge in precision cancer therapy, contributing to treatment resistance, therapeutic failure and poor prognosis [1,2]. With the growing use and reducing cost of next generation sequencing, the full extent of the complexity and genomic diversity within tumors are becoming more apparent [1,3,4]. Genotype-directed targeted therapies are becoming the standard of care, and as tumor molecular profiling becomes more widely used in routine practice, physicians will require the necessary tools to translate genomic information into clinically actionable results. The consequences of intratumor heterogeneity, such as resistance to drug therapy [3,4,5] leading to disease recurrence and death, is at least partially the result of limitations in the ability to define the clonal frequency of driver events for prioritization of drug targeting in tumors. Furthermore, it has been demonstrated that high levels of ITH results in poorer survival outcomes across a wide range of cancer types [6,7]. To mitigate this challenge, a more comprehensive view of the mutational diversity of each tumor lesion is required. 

The mutational diversity attributed to ITH limits our ability to resolve the full spectrum of cancer pathway aberrations through a single biopsy of the tumor lesion and may under/overestimate driver alterations [4,8,9]. Therefore, multi-region tumor biopsies (MRTBs) are highly beneficial to attenuate the challenge of estimating the prevalence of oncogenic clonal driver mutations. Targeting clonal driver (truncal) mutations would potentially be more effective than targeting subclonal (branch) mutations in a tumor [10,11]. Yap et al. proposed the targeting of genetic alterations located on the trunk of an individual’s phylogenetic tree as a more effective clinical strategy [10] as truncal mutations are more likely to represent the core driver mutations within the tumor [10]. In view of the importance of identifying truncal mutations, in silico approaches such as the ABSOLUTE algorithm [12] have been developed to predict truncal variants from a single biopsy sample. However, their ability to identify actionable truncal mutations that would be clinically relevant is hitherto unknown. Similarly, various gene panels have been utilized for diagnostic purposes but the minimum number of MRTB samples needed to address issues associated with ITH remains unknown. This study—conducted across six major cancer types—aims to outline: (a) the safety and significance of MRTB to help navigate the complexities of ITH, (b) the minimum number of MRTB samples required when different gene panels were used for clinical assessment, and (c) the feasibility and clinical efficacy of the approach for identifying clinically actionable truncal mutations (i.e., mutations present in all MRTB obtained from a single tumor lesion) and their outcomes when targeted for strategic therapeutic intervention.

## 2. Results

**Figure 1:**
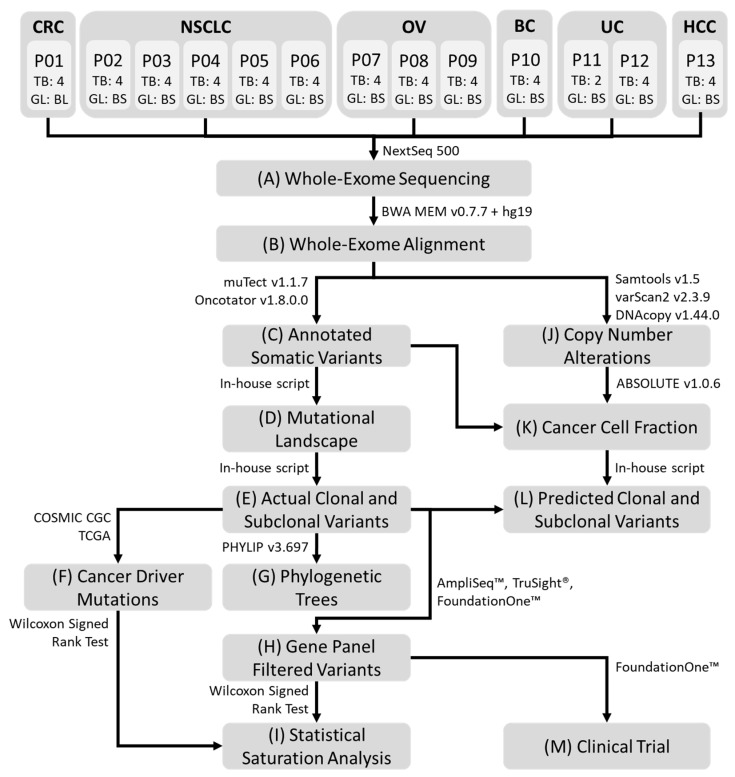
Representative workflow of the processing pipeline. (**A**) Whole-exome sequencing was performed on all germline and MRTB samples obtained from each patient. Bioinformatics analysis was subsequently performed: (**B**) alignment of sequence reads; (**C**) somatic variant calling and variant annotation; (**D**) generation of non-synonymous somatic mutational landscape across all patients; (**E**) identification of truncal and branch variants present in each patient; (**F**) curation of statistically significant somatic cancer driver mutations; (**G**) construction of phylogenetic trees from non-synonymous somatic variants; (**H**) filtering of genetic variants using AmpliSeq™, TruSight® and FoundationOne™ cancer gene panels; (**I**) statistical saturation analysis to determine the minimum number of MRTB samples needed (to alleviate challenges associated with ITH) in relation to the gene panel used; (**J**) copy number alterations analysis; (**K**) estimation of cancer cell fraction (CCF); (**L**) prediction of putative truncal variants using two different threshold metrics, namely variant allele frequency and CCF; (**M**) informed targeted therapies were performed based on patients’ mutational profile that reflects genes from the AmpliSeq™ cancer gene panel. MRTB: multi- region tumor biopsy; ITH: intratumor heterogeneity; TB: the number of MRTB samples resected from the patient; GL: the type of germline sample; BL: whole blood sample; BS: buccal swab sample. CRC: Colorectal cancer; NSCLC: Non-small cell lung cancer; OV: Ovarian Cancer; BC: Breast cancer; UC: Uterine Cancer; HCC: Hepatocellular Carcinoma; P: Patient.

A cohort of 15 patients with metastatic colorectal carcinoma (CRC; *n* = 1), non-small cell lung cancer (NSCLC; *n* = 6), ovarian carcinoma (OV; *n* = 3), breast carcinoma (BC, *n* = 1), uterine carcinoma (UC, *n* = 2), hepatocellular carcinoma (HCC; *n* = 1), or cervical cancer (CC, *n* = 1) were recruited to the study. A single-pass radiologically-guided percutaneous biopsy technique was used to obtain MRTBs from a dominantly-progressing metastatic lesion in each patient with core biopsies taken at least 2 mm apart within the same metastatic lesion. Two patients (one with NSCLC and another with cervical cancer) were excluded from analysis as all the MRTB samples collected from them failed quality control (QC). One patient (UC) (P11; Figure 1) had two (out of four) biopsy samples that failed QC which were subsequently excluded from the analysis. Similarly, one patient’s (P01) germline sample (i.e., buccal swab) failed QC and was replaced with whole blood sample. All 15 patients tolerated the procedure well with no significant adverse events, except for one patient (P09) who developed a moderately-sized right sided pneumothorax requiring observation overnight and serial imaging to ensure spontaneous resolution of the pneumothorax.

The investigation pipeline adopted for analyzing whole-exome sequencing (WES) data that passed QC is illustrated in Figure 1. All processed samples had a DNA concentration greater than 4 ng/μL. Average sequencing depth, Q30 percentage and uniformity of coverage obtained were 128X ± 29.2, 87.5% ± 4.35, and 91.2% ± 1.94 respectively.

### 2.1. Tumour Variant Load

**Figure 2:**
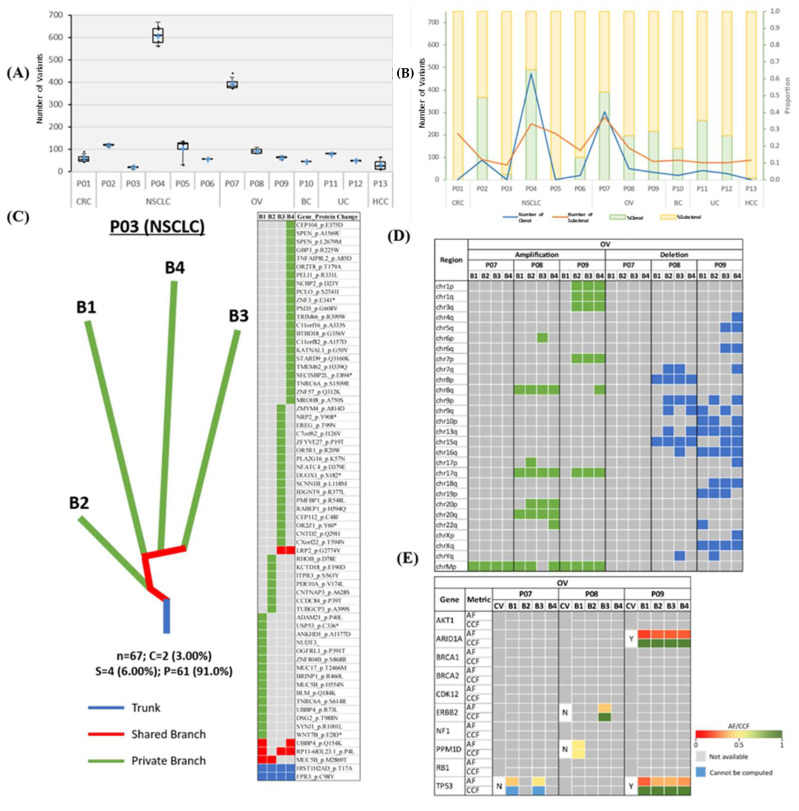
Mutational landscape of patients across six cancer types. (**A**) Boxplot illustrating nssML. A cross (+) represents the mean value of the data. (**B**) Line chart and stacked bar chart representing the number and proportion of truncal/branch variants, respectively. (**C**) Representative phylogenetic tree and mutation heatmap for patient P03. Trunk, branch and private branches of the tree signify mutations that occur in all, in some but not all, and only one MRTB sample(s) resected from the patient, respectively. Heatmap demonstrates the presence (green: private; red: branch; blue: trunk) or absence (gray) of NSS mutations in each MRTB sample. Bx denotes an MRTB sample with identification number x. The total number of NSS, truncal (percentage), branch (percentage), and private (percentage) mutations are denoted by ‘n’, ‘C’, ‘S’, and ‘P’, respectively. (**D**) Heatmap illustrating the presence and absence (gray) of CNAs for patients with OV. Large-scale amplifications and deletions are represented with areas filled with green and blue, respectively. (**E**) ssCDMs for OC and their associated AF and CCF. CV: clonal (truncal) variant; Y: yes; N: no; AF: allele frequency; CCF: cancer cell fraction.

The mutational landscape of each patient was examined to evaluate the extent of ITH across different cancer types. The non-synonymous somatic mutational load (nssML)—defined as the total number of non-synonymous somatic (NSS) variants present—was scrutinized for each biopsy sample (Figure 2A). Results indicate that patient P04 has the highest average non-synonymous somatic mutational load (608.0 ± 41.7), while patient P05 with NSCLC has the highest diversity (i.e., difference in non-synonymous somatic mutational load) among the four MRTB samples analyzed (104.3 ± 49.3). The median diversity across all patients was 7.46 (range: 0.957 to 49.3). Friedman test of difference among the different MRTB samples indicated no statistically significant difference between the number of non-synonymous somatic variants present in each MRTB sample (*p* = 0.691).

### 2.2. Intratumor Heterogeneity

To investigate the extent of the intratumoral heterogeneity, the amount of truncal (i.e., ubiquitous non-synonymous somatic variants that occur in all MRTB samples analyzed) and branch (i.e., non-synonymous somatic variants that do not occur in all MRTB samples) variants were analyzed (Figure 2B). Phylogenetic trees were also constructed to illustrate this phenomenon graphically (Figure 2C, Figure S1). As demonstrated, two patients (P01 and P05) did not have any truncal variants while patients P03 and P13 only had two and one truncal variant(s), respectively. On average, 24.1% ± 20.7, 14.7% ± 13.7 and 61.2% ± 20.6 of non-synonymous somatic variants were truncal, branch and private mutations, respectively. A high level of intratumoral heterogeneity (75.5% ± 34.6) across different tumors was observed, with private mutations dominating the mutational landscape (*p* < 0.05). When copy number alterations (CNAs) were interrogated, a moderate degree of diversity (branch amplification: 54.3% ± 34.7, *p* = 0.083; branch deletion: 59.4% ± 34.8, *p* = 0.050) was observed (Figure 2D, Figure S2).

Statistically significant somatic cancer driver mutations (ssCDMs) were juxtaposed with non-synonymous somatic variants identified in our study cohort. Results indicate that detectable somatic cancer driver mutations were more likely to be truncal variants (68.2%; Figure 2E, Figures S3 and S4); however, the difference was not statistically significant (*p* = 0.177). Truncal somatic cancer driver mutations across this study cohort include *AKT1, ATM, BCOR, CHD4, KRAS, MAP3K1*, and *PIK3CA*; conversely, branch somatic cancer driver mutations include *ERBB2, FOXA1*, and *PPM1D*. In our cohort, *EGFR* mutations were confined to lung cancers, with three out of four (75%) NSCLC patients found to harbor a truncal variant in at least one reportable mutation in *EGFR* [13].

### 2.3. Statistical Saturation Analysis

The relationship between truncal variants and the number of MRTB samples analyzed was examined. A unique variant in this case refers to a distinct non-synonymous somatic variant that appears in at least one of the MRTB samples analyzed simultaneously. In general, with an increasing number of MRTB samples, a monotonically increasing trend in the number of unique variants and correspondingly decreasing number of truncal variants can be observed (Figure S5). 

At the exome level (i.e., WES NSS gene panel), on average 31.1 ± 5.12 more unique non-synonymous somatic variants were detected with every additional MRTB sample analyzed. Conversely, using the number of MRTB samples analyzed simultaneously as the baseline reference to determine putative truncal variants (PTVs), a monotonically decreasing number of PTVs can be observed with an increasing number of MRTB samples. The number of PTVs dropped by 16.1 ± 17.9 on average with every additional MRTB sample, with the decrease most pronounced (36.8 ± 19.7) when two MRTB samples were analyzed compared to just one. Similar trends were observed from filtered variants when the WES data was mapped to genes matching four cancer gene panels—namely COSMIC Cancer Gene Census (CGC), Ion AmpliSeq™ Cancer Hotspot Panel v2 (Life Technologies, Carlsbad, CA, USA), TruSight® Cancer panel (Illumina Inc, San Diego, CA, USA) and FoundationOne™ cancer gene panel (Foundation Medicine, Cambridge, MA, USA) (Figure S5, Tables S1 and S2). However, the change in the number of unique/truncal mutations with increasing MRTB samples was less pronounced (<2 variants on average).

**Figure 3:**
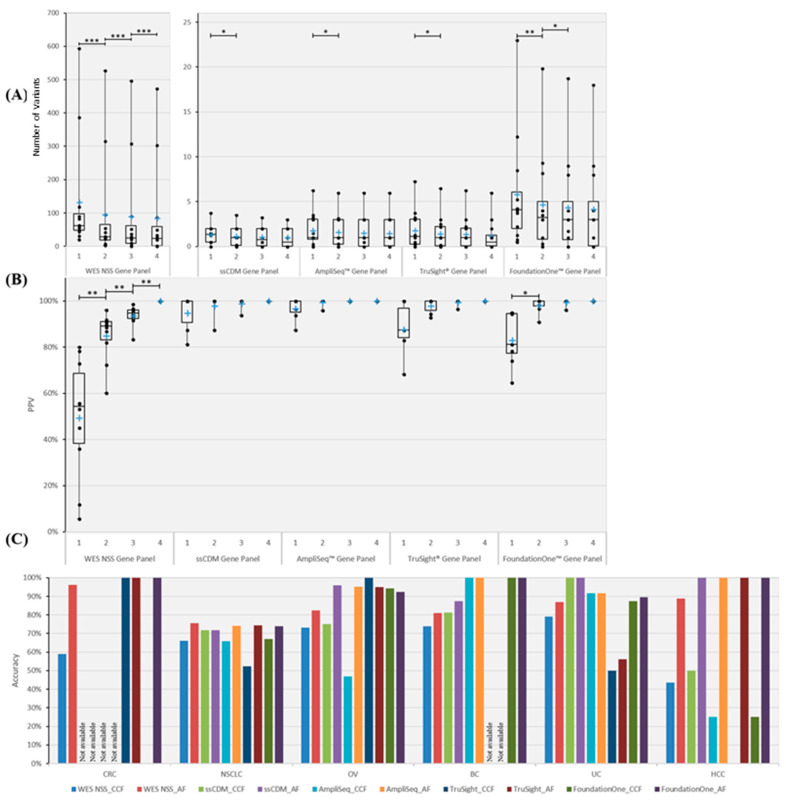
Number, PPV and in silico prediction accuracy of truncal variants across different gene panels. Five gene panels were scrutinized, namely WES NSS, CGC, AmpliSeq™, TruSight® and FoundationOne™ cancer gene panels. (**A**) Boxplot illustrating the number of PTVs across different numbers of MRTB samples analyzed concurrently. (**B**) PPV of PTVs in relation to the number of MRTB samples interrogated simultaneously. (**C**) Best average prediction accuracy of PTVs across different cancer types. Two types of thresholds were used to classify variants into either truncal or branch, namely AF and CCF. Based on the respective threshold, the best average prediction accuracy achievable (within the defined search domain) among all patients with the same cancer type (across different gene panels) is portrayed above. A single asterisk (*) denotes *p* < 0.05, double asterisks (**) signify *p* < 0.01, while triple asterisks (***) indicate *p* < 0.001. A cross (+) represents the mean value of the data. ‘Not available’ signifies that no variants that are associated with the specific gene panel were found.

To quantitatively corroborate the minimum number of MRTB samples required across different gene panels, statistical saturation analysis was conducted. Results (Figure S6) indicate that every additional MRTB sample analyzed increases the ability to detect unique variants when WES non-synonymous somatic, TruSight® and FoundationOne™ gene panels were used; as for CGC and AmpliSeq™ gene panels, at least two or three MRTB samples (depending on the panel used) were required, respectively, before changes in the number of unique variants became statistically not significant. To identify PTVs, results (Figure 3A) suggest that at least two MRTB samples were required for the CGC, AmpliSeq™ and TruSight® gene panels, and three samples were required for the FoundationOne gene panel; the WES NSS gene panel, on other hand, required four or more MRTB samples based on our analysis. In addition, the positive predictive value (PPV) was determined to evaluate the extent to which truncal variants (defined using four MRTB samples as the baseline reference) can be identified among all variants found in a set of less than four MRTB samples. Results (Figure 3B) indicate that four (or more) and two MRTB samples are needed for WES NSS and FoundationOne™ gene panels, respectively, while CGC, AmpliSeq™ and TruSight® cancer gene panels only require a single biopsy sample. The greatest significant increase in PPV based on WES NSS and FoundationOne panels were from one analyzed sample to two samples (Figure 3B).

### 2.4. Prediction of Truncal Mutations

To evaluate the ability to identify truncal variants (defined based on four MRTB samples) using a single biopsy sample, two metrics were used as classification thresholds, namely allele frequency (AF) and cancer cell fraction (CCF). Computation of CCF values—the proportion of cancer cells within which the variant is present—for patient P07 (OV) was unable to be performed due to inadequate information related to somatic copy number alteration. Hence, patient P07 was excluded for the purpose of this analysis.

First, the threshold value (for each respective metric) that produces the best average prediction accuracy across all patients was examined. Results, as illustrated in Figure S7, suggest that AF generally outperformed CCF across different patients. Average prediction accuracy improved between 2.7% and 15.3% (across different gene panels) when AF was used as the classification threshold. However, statistical significance of difference was achieved for the WES NSS gene panel only (*p* = 0.021).

Next, the threshold value (for each respective metric) that produces the best average prediction accuracy across patients with the same cancer type was scrutinized. Results, as demonstrated in Figure 3C, show that AF outperformed CCF by 15.6% to 30.4% across the different gene panels, with the FoundationOne™ cancer gene panel having the largest difference. Statistical significance of difference was achieved for the WES NSS gene panel only (*p* = 0.031).

### 2.5. Clinical Therapeutic Intervention

Three (23.1%) patients (P06, P10, and P11) received an inhibitor targeting an actionable truncal mutation based on molecular profiling while another six (46.2%) patients were treated with non-actionable truncal mutation-directed therapy either because they did not have any actionable truncal mutations or there was no available therapy to target the actionable truncal mutation at our center (Table 1). An illustrative example of the patients’ mutational profile can be found in Figures S8 and S9.

Using each patient as his/her own control as a strategy to attenuate confounding factors resulting from the diverse patient population and tumor types, we assessed the clinical efficacy of actionable truncal mutation-directed therapy by comparing progression free survival (PFS) on actionable truncal mutation-directed therapy (PFS-actionable truncal mutation-directed therapy) or non-actionable truncal mutation-directed therapy with the PFS for the most recent prior therapy (PFS-A) in each of these patients [14]. Two NSCLC patients harboring an Epidermal growth factor receptor (EGFR)_T790M mutation were treated with a single agent EGFR_T790M specific tyrosine kinase inhibitor [15] with differing clinical outcomes. Patient P06 had a truncal EGFR_T790M mutation while patient P05 had an EGFR_T790M mutation as a branch mutation. Patient P06 had a partial response and was still on active treatment at last review with a PFS of >25 months (Figure S10a), while patient P05 developed worsening neuro-cognitive defects resulting in cessation of treatment after two months. The PFS ratio for patients P05 and P06 was 0.06 and 10.2, respectively.

Patient P10 with breast cancer and a phosphoinositide-3-kinase catalytic alpha polypeptide (PIK3CA)_H1047R truncal mutation was enrolled into a highly selective PI3Kα/β inhibitor phase 1 (dose escalation) trial [16], but progressed shortly after with a PFS of 1.9 months and PFS ratio of 0.95 despite deriving symptomatic benefit while on the trial. Lastly, patient P11 with uterine carcinoma harbored a truncal RAC-alpha serine/threonine-protein kinase (AKT1) E17K mutation in two out of two of her MRTB cores analyzed (only two of four cores had DNA of sufficient quality for analysis in her case). She had significant sacral bone pain from bone metastasis and received a pan-AKT inhibitor. Strikingly, her pain significantly improved and subsequent scans revealed a 21% reduction in the sum of target lesions with a PFS of 6.1 months (Figure S10b). When compared to the PFS from her most recent physician’s choice therapy, a PFS ratio of 1.5 was observed.

It is noteworthy that the CCF metric performed relatively well in predicting the truncal status of the variants targeted. The median PFS-actionable truncal mutation-directed therapy for the small number of patients treated with actionable truncal mutation-directed therapy was 6.1 months, with a median PFS ratio of 1.5. These findings do suggest that the truncal status of tumors influences response and, if validated, could potentially be used in personalized cancer treatment to help prioritize therapeutic strategies.

## 3. Discussion

ITH represents a significant challenge to precision medicine and contributes to drug resistance. Several studies employing multi-region tumor sampling from post-surgical samples have greatly increased our understanding of tumor evolution and highlighted the importance of tumor sampling from spatially distinct areas in order to avoid erroneous interpretation of genomic data from single sampling bias [8,9,10]. In clinical practice, however, a systematic regional analysis of resected tumor specimens is unfeasible in the majority of patients with metastatic or recurrent cancer who may only have limited accessible intracorporeal tissue for sampling/biopsy. Hence, high quality patient samples across six major cancer types were analyzed to address certain exigent issues related to ITH and devise a potential novel solution to tackling the complexities of tumor heterogeneity when confronted with the reality of treatment decision-making based on limited access to tumor tissue. 

Results in our small cohort demonstrate a high degree of ITH (>65% branch mutations) across the majority of patients, with private mutations dominating the mutational landscape. Clearly, this indicates that ITH is a ubiquitous issue that would confound the ability to identify bona fide truncal variants. Statistical saturation analysis demonstrates that for small targeted cancer gene panels like CGC, AmpliSeq™ and TruSight®, a minimum of two MRTB samples are required to identify PTVs; for a larger cancer gene panel like FoundationOne™, at least three MRTB samples are needed. The determination of the minimum number of MRTB samples required is highly valuable as it enables clinicians to find the equilibrium between cost and accuracy (of identifying bona fide truncal variants), and allows the choice of which cancer gene panel to use with the amount of tumor tissue available.

Examination of the nssML of each patient indicates that individual intratumor biopsy samples comprise a similar amount of NSS variants (Figure 2A) while the aggregated nssML shows that every additional MRTB sample would offer a statistically significant increment in the total number of NSS variants (Figure S6). Correlation analysis indicates a strong correlation between the average number of NSS variants among individual intratumor biopsy samples and aggregated NSS variants across all four MRTB samples (Pearson’s rho = 0.975, *p* < 0.001). This suggests minimal intratumoral variation in the ML based on our series and that mutational burden is less likely to be impaired by sampling bias. 

Given the coveted utopia of making informed clinical decisions based on a single biopsy sample, in silico methods for predicting truncal variants are of particular interest. AF and CCF are two favored metrics commonly used. Empirical experiments indicate that when AF was used as the threshold to classify truncal variants, it achieved comparable, if not better, accuracy compared to CCF; although both approaches were less compelling for some cancer types. Of note, different cancer types (at the whole-exome level) favor different threshold values for segregating truncal from branch variants, suggesting that each tumor type exhibits distinct biological characteristics that require dedicated data analytics.

Improved clinical outcomes were observed in two out of three patients whose truncal mutations were selectively targeted. Remarkably, all patients in our series treated with actionable truncal mutation-directed therapy derived symptomatic benefit with improvement in their performance status. The small patient numbers across a diverse spectrum of tumors limits our ability to draw significant conclusions within each tumor type, but nonetheless demonstrates its applicability in a variety of tumor types and preliminary evidence of clinical benefit when used for therapeutic prioritization in selected patients. It is noteworthy that patient P05—who had an EGFR_T790M branch mutation that was targeted—did not respond well to the treatment (Table 1). This reaffirms the hypothesis that increased therapeutic efficacy can be achieved by targeting truncal mutations within a tumor, and that targeting branch mutations may result in only partial treatment efficacy and/or accelerated growth in non-targeted subpopulations [17]. Undeniably, the cost per patient of this approach is high; it has been estimated at USD$5000 per patient for the acquisition of biopsy samples and profiling of four biopsy core samples as well as a germline control, but this is likely to be mitigated in the future as next generation sequencing technologies become more widely used and cost of sequencing gradually decreases. Crucially, the data provided by multi-region sequencing of a tumor could have important implications for the prioritization of druggable targets in the clinical setting. To the best of our knowledge, this is the first study assessing the feasibility and utility of obtaining tissue biopsies from multiple spatially separated regions from a single metastatic site percutaneously. A limitation of this study is the small sample size. Nevertheless, it provides adequate resolution into the complexity and management of ITH. In addition, the ITH analysis performed in this study is based on the construction of phylogenetic trees with the implicit assumption that a tumor sample can be meaningfully summarized as the collection of mutations observed in that sample, or that only a single or dominant clone exists per sample that carries all mutations, which could lead to biased inferences.

In our study, we only analyzed single nucleotide variants (SNV) which may potentially underestimate the frequency of clinically actionable mutations and the mutational load of the tumor. We focused solely on SNV mainly because they make up the majority of pathogenic variants relevant in solid tumor malignancies (59.39%) compared to other genomic alterations such as indels, structural variants and copy number loss [18]. Indeed, the majority of annotated variants in oncogenic and actionable target databases such as OncoKB and cancer hotspots consist of predominantly SNV. In addition, the majority of approved targeted inhibitors available for solid cancers currently are also mainly directed at aberrations associated with SNV. As our results relied entirely on WES analysis, and so one of the limitations of our study is the dependence on the size of the panel testing. As the number of variants being considered increases, so does the required number of samples. Our study also used fresh frozen tissue for WES analysis, which resulted in 13 of the 81 samples collected failing quality assurance due to degradation of DNA. As we continue to expand our taxonomy of tumors and seek to enhance the applicability of this approach in clinical practice, it would be ideal to optimize this approach for the clinical grade analysis of formalin-fixed, paraffin-embedded tumor samples, to enable histological and immunohistochemical analyses of samples to be performed in parallel with genomic analysis in the future. It has been suggested that liquid biopsies based on genomic analyses of circulating cell-free tumor DNA (ctDNA) and circulating tumor cells (CTC) may obviate the need for tumor biopsies [19]. Liquid biopsy platforms offer the potential for real-time sampling and resampling of tumor material for monitoring of therapeutic efficacy [20] and early detection of resistance subclones [21]. Furthermore, the MRTB approach we have used in this study will not be feasible in patients with inaccessible lesions. However, the inability to characterize liquid biopsies histologically limits the extent of biomarker analyses, particularly where tumor microenvironmental features (e.g., programme cell death-1/programme cell death ligand-1(PD1/PDL1) protein expression), immune cell infiltrates and stromal content are concerned. Further studies comparing the clinical utility of multi-spatial or multi-lesional biopsy approaches with that of liquid biopsies in monitoring the emergence of resistance and therapeutic efficacy are eagerly awaited

## 4. Materials and Methods

### 4.1. Patients and Specimens Collection

All patients were recruited and treated at the National University Cancer Institute (NCIS), Singapore, between December 2014 and May 2016. WES and data analytics were performed at the National University of Singapore (NUS), Singapore. All procedures were conducted in accordance with the approved protocols and written informed consent was provided by the patients (File S1). Eligible patients were at least 21 years of age, had a histological or cytological diagnosis of advanced or metastatic solid malignancy and had recurrent disease for which tissue biopsy was indicated as part of routine clinical practice. This study was approved by the National Health Group Domain Specific Review Board IRB number 2014/00665

Each patient had 5 tumor biopsy samples obtained from one metastatic lesion using a single-pass radiologically-guided percutaneous biopsy technique. This technique involves the insertion of a coaxial needle together with its trocar into a lesion. The trocar is subsequently removed to allow for the introduction of a biopsy device that is composed of a needle with a 1.5 cm throw to facilitate multiple passes along acute angles from a single lesion via a single percutaneous access. Each biopsy sample was obtained at least 2 mm apart. One biopsy core was sent to the histopathology lab as part of routine clinical management while the remaining four tumor biopsy samples were analyzed using WES. Four patients had biopsies obtained from the lung (P3, P4, P5 and P6), four had peritoneal nodes biopsied (P1, P7, P8 and P9), three patients underwent a liver biopsy (P10, P11 and P12) and one patient each had a bone (P13) and supraclavicular lymph node (P2) biopsied. Germline samples were collected from each patient in the form of a buccal swab. If the germline sample failed quantitative QC, whole blood would be used as replacement. Samples with DNA concentration <4 ng/μL would be deemed to have failed QC. Radiological images were obtained as part of clinical care. 

### 4.2. Whole-Exome Sequencing

Extraction of genomic DNA from tumor samples was carried out using the Qiagen Allprep DNA/RNA Micro Kit (Qiagen, Hilden, Germany). The MasterAmp Buccal Swab Kit (Epicenter, Madison, WI, USA) was used to extract DNA from buccal swab samples while the Qiagen EZ1 DNA Blood 350 µL Kit (Qiagen, Hilden, Germany) was used to process whole blood samples. An Illumina NextSeq 500 Sequencing System (Illumina, San Diego, CA, USA) was utilized to perform 150 base pair paired-end WES. All experiments were conducted in accordance to manufacturer guidelines at the Cancer Science Institute of Singapore, NUS. Full patient data can be found in the National Centre for Biotechnology Information (NCBI) Sequence Read Archive (SRA) under accession number SRP137039

### 4.3. Sequencing Reads Alignment and Somatic Variant Detection

Sequence reads were aligned to the hg19 reference genome using the Burrows–Wheeler Aligner (BWA) v0.7.7 [22] and realignment to the hg19 reference genome was performed by using the Genome Analysis ToolKit (GATK) v3.3.0 [23]. Variant calling was performed with duplication removal and base recalibration prior to variant calling using MuTect somatic variant caller v1.1.7 [24] and annotated using Oncotator v1.8.0.0 [25]. Only NSS variants were filtered out for analysis independent of CCF or AF. All sequencing data have been made available in the NCBI Sequence Read Archive (SRA) under accession number SRP137039.

### 4.4. Copy Number Alterations

Somatic CNAs were detected using several algorithms. Succinctly, the computation of raw copy number calls and the adjustment of GC content of the raw copy number calls were performed using VarScan2 v2.3.9 [26]. Re-centering and segmentation of the adjusted copy number calls were conducted using DNAcopy v1.44.0 [27]. Sample purity was assessed using the ABSOLUTE algorithm (Appendix A, Table A1).

### 4.5. Cancer Gene Panels

Five gene panels—WES NSS, COSMIC Cancer Gene Census (CGC), the Ion AmpliSeq™ Cancer Hotspot Panel v2, the TruSight® Cancer panel and the FoundationOne™ cancer gene panel—were examined. The WES NSS gene panel comprises of all protein-coding genes (with NSS property) in the genome, while the CGC gene panel consists of all statistically significant cancer-specific genes curated from the COSMIC Cancer Gene Census (CGC) [28,29] and The Cancer Genome Atlas (TCGA) [30,31]. The complete list of CGC interrogated genes for CRC (*n* = 29), NSCLC (*n* = 30), OV (*n* = 10), BC (*n* = 52), EC (*n* = 58), and HCC (*n* = 26) is available in Figures S3 and S4. Commercial cancer gene panels like AmpliSeq™, TruSight® and FoundationOne™ are comprised of 50, 94, and 315 cancer-related genes, respectively; their interrogated gene lists are available at ThermoFisher [32], Illumina [33], and Foundation Medicine [34], respectively. 

All NSS variants identified in the WES NSS gene panel were subsequently juxtaposed with individual targeted cancer gene panels (i.e., CGC, AmpliSeq™, TruSight® and FoundationOne™) and variants from mismatched genes were winnowed out. The resulting list of variants for each cancer gene panel was used for subsequent downstream analysis.

### 4.6. Construction of Phylogenetic Trees

The construction of phylogenetic trees was carried out based on a binary table that represents the presence or absence of variants across all MRTB samples. Using the PHYLogeny Inference Package v3.695 (PHYLIP) [35] and matched germline information as the outgroup root, discrete character parsimony was used to generate the topology of the phylogenetic trees. Based on the computed mutation counts, the length of the trunk, shared and private branches were drawn accordingly.

### 4.7. Statistical Saturation Analysis

The computation of the average number of unique variants present when ‘k’ number of MRTB samples were analyzed concurrently was performed based on the following Formula (1):

(1) $$Average\;unique\;variants_{k}=\frac{1}{n}\sum_{i=1}^{n}x_{i},$$

where ‘n’ denotes the number of permutation combinations available when ‘k’ number of MRTB samples were selected from 4 MRTB samples, and ‘x_i_’ refers to the number of variants that are present in at least one of the MRTB samples examined in combination set ‘i’. Similarly, the average number of PTVs was calculated based on the formula above but with ‘x_i_’ defined as the number of variants present in all MRTB samples scrutinized in combination set ‘i’. 

PPV, on other hand, was computed based on the following Formula (2):

(2) $$Average\;PPV_{k}=\frac{1}{n}\sum_{i=1}^{n}\frac{C}{PCV_{i,k}},$$

where ‘n’ denotes the number of permutation combinations available when ‘k’ number of MRTB samples were selected from 4 MRTB samples, ‘C’ represents the number of variants that occur in all 4 MRTB samples, and ‘PTV_i,k_’ (i.e., putative truncal variant) refers to the number of variants present in all ‘k’ number of MRTB samples examined simultaneously in combination set ‘i’.

All statistical hypothesis tests were conducted using the Wilcoxon signed rank test unless otherwise stated. Statistical significance is considered when the *p*-value is less than 0.05.

### 4.8. Cancer Cell Fraction and Allele Frequency

The CCF value was estimated using the ABSOLUTE algorithm [13] for each somatic single nucleotide variant (SNV) site based on its AF, CNAs, ploidy and purity of the tumor tissue analyzed. Based on the computed CCF values, a range of thresholds—from 0.90 to 1.00 incremented at a step size of 0.01—were used to classify variants into either truncal or branch.

AF was calculated by dividing the number of alternative sequence read counts with the total number of (alternative and reference) sequence read counts. Likewise, the clonality of variants was determined by comparing the variant’s AF with a range of thresholds (from 0.01 to 0.55 incremented at a step size of 0.01). The respective thresholds used for both the cancer cell fraction and allele frequency analysis according to the individual panels are shown in Table 2 below:

### 4.9. Prediction of Truncal Mutations

To stratify variants into either truncal or branch based on a single biopsy sample, different (AF and CCF) threshold values were investigated. For each threshold value examined, the following formula was employed to compute the average classification accuracy, by which 4 MRTB samples were used as the baseline reference for defining bona fide truncal variants (3).

(3) $$Average\;Accuracy_{k}=\frac{1}{4}\sum_{i=1}^{4}\frac{x_{i,k}}{y_{i}},$$

where ‘k’ denotes the examined threshold value, ‘x_i,k_’ represents the number of correct classification made for biopsy sample ‘i’ using threshold value ‘k’, and ‘y_i_’ refers to the total number of variants assessed for biopsy sample ‘i’.

### 4.10. Assessing Targeted Therapy Outcomes

Treatment decisions were made using molecular profiling results from one core biopsy, as reported in the Intergrated Molecular Analysis of Cancer (IMAC) study [14] while the remaining four core biopsies were analyzed for this study. Using each patient as his/her own control as a strategy to attenuate confounding factors resulting from the diverse patient population and tumor types, we assessed the clinical efficacy of actionable truncal mutation-directed therapy by comparing the PFS for each patient who received actionable truncal mutation-directed therapy (PFS-actionable truncal mutation-directed therapy) with the PFS for the therapy immediately before actionable truncal mutation-directed therapy (PFS-A) [36]. If the PFS of PFS-actionable truncal mutation-directed therapy/PFS-A ratio was ≥1.3, then the molecular profiling-selected actionable truncal mutation-directed therapy was defined as having benefit for the patient compared to the physician’s choice chemotherapy. The PFS ratio was defined as $\frac{\mathrm{PFS}–\mathrm{actionable}\;\mathrm{truncal}\;\mathrm{mutation}–\mathrm{directed}\;\mathrm{therapy}}{\mathrm{PFS}\;\mathrm{for}\;\mathrm{the}\;\mathrm{therapy}\;\mathrm{immediately}\;\mathrm{before}\;\mathrm{actionable}\;\mathrm{truncal}\;\mathrm{mutation}–\mathrm{directed}\;\mathrm{therapy}\;\left(\mathrm{PFS}–A\right)}$ and was used to evaluate the efficiency of the therapeutic intervention [36].

## 5. Conclusions

In conclusion, this study has demonstrated: (i) the importance of performing multiple biopsies despite extant in silico prediction methods, (ii) the minimum number of MRTB samples required to alleviate challenges related to ITH is dependent on the tested hypothesis and the examined gene panel, but that at least two biopsies should be submitted for analysis to achieve a PPV of >90% identifying AT mutations, and (iii) the feasibility and clinical efficacy of adopting the proposed approach for strategic therapeutic intervention. Further validation of this approach for identifying and targeting AT mutations in larger cohorts will be required to fully assess its potential value as a precision medicine strategy to circumvent the challenges of intratumoral heterogeneity in cancer therapy.

## Figures and Tables

**Table 1:** Clinical details of patients who received therapy targeting their actionable truncal mutation.

Cancer Type	Patient	Age	Sex	No. of MRTB Samples with Abnormality of Interest	No. of MRTB Samples that CCF Metric Classified as Clonal	Targeted Abnormality	Therapeutic Intervention	PFS (Months)		PFS Ratio	Radiological RECIST (v1.1) Response
								Initial Therapy	Actionable Truncal Mutation-Directed Therapy		
**NSCLC**	P06	74	M	4/4	3/4	EGFR T790M	T790M inhibitor	2.5	25.5	10.2	PR
**NSCLC**	P05	43	M	3/4	NA	EGFR T790M	T790M inhibitor	2.1	3.6	1.71	SD
**BC**	P10	41	F	4/4	4/4	PIK3CA H1047R	PI3Kα/β inhibitor	2	1.9	0.95	PD
**UC**	P11	46	F	2/2	2/2	AKT1 E17K	pan-AKT inhibitor	4	6.1	1.53	SD

M: male; F: female; SD: stable disease; PR: partial response; PD: progressive disease (based on RECIST v1.1); PFS: progression free survival. RECIST: Response evaluation criteria in solid tumors; EGFR: Epidermal growth factor receptor; PIK3CA: phosphoinositide-3-kinase catalytic alpha polypeptide; AKT: RAC-alpha serine/threonine-protein kinase.

**Table 2:** Thresholds used to determine the clonality of the variants.

Panel	CCF	AF
FoundationOne	0.92	0.13
AmpliSeq	0.92	0.15
TruSight	0.96	0.13
WES	1	0.16

## Supplementary Materials

The following are available online at https://www.mdpi.com/2072-6694/12/6/1599/s1, Figure S1: Phylogenetic trees constructed from NSS mutations, Figure S2: Heatmap visualization illustrating the presence/absence of copy number alterations in relation to each region, Figure S3: ssCDMs for CRC, NSCLC, and OV, Figure S4: ssCDMs for BC, UC, and HCC, Figure S5: Mutational characteristics across different number of MRTB samples and gene panels, Figure S6: Boxplot illustrating the average number of unique variants across different gene panels and number of MRTB samples, Figure S7: Best average prediction accuracy of PTVs across different patients, Figure S8: Illustrative example of patients P01-P06 mutational profile, Figure S9: Illustrative example of patients P07-P13 mutational profile, Figure S10: Pre - treatment and 8- week post treatment images from, Table S1: Average number of unique variants detected across different number of MRTB samples and gene panels, Table S2: Average number of PTVs detected across different number of MRTB samples and gene panels, File S1: Study Protocol.

## Appendix A

**Table A1:** Assessment of tumor purity for each sample using ABSOLUTE algorithm. Abbrev: VAF, variant allele frequency; BRCA, breast carcinoma; CRC, colorectal carcinoma; OV, ovarian carcinoma; UCEC, uterine carcinoma; LIHC, hepatocellular carcinoma; NaN, not computable.

Sample	Patient	Patient label	Disease	ABSOLUTE_call Status (Called = Clonal)	ABSOLUTE_purity	ABSOLUTE_ploidy	ABSOLUTECancer DNA Faction	ABSOLUTECoverage for 80% Power	VAF Range-All Variants	VAF Range-Putative Truncal Variants	VAF Range-Private Variants	VAF Range-Branch Variants	Mclust.wCN.Cluster#	Mclust.noCN.Cluster#	VAF_ROC_AUC_TruncalVsNonTruncal	VAF_YoudenThreshold_TruncalVsNonTruncal	VAF_YoudenThreshold_TruncalVsNonTruncal_Accuracy	Mclust.cn.threshold_clusterWHighestVAF	Mclust.cn.threshold_TruncalVsNonTruncal_Accuracy	Remark
RE3F1	RE3	P01	CRC	called	0.25	5.86	0.49	64	0.14038	NaN	0.135531915	0.2446	2	2	NaN	NaN	NaN	0.241285714	NaN	No Truncal Mutation detected
RE3F2	RE3	P01	CRC	called	0.21	1.94	0.2	52	0.134576087	NaN	0.099253968	0.152966942	1	2	NaN	NaN	NaN	0.1086	NaN	No Truncal Mutation detected
RE3F3	RE3	P01	CRC	called	0.28	4.02	0.44	49	0.146770833	NaN	0.143824176	0.2004	2	2	NaN	NaN	NaN	0.348666667	NaN	No Truncal Mutation detected
RE3F4	RE3	P01	CRC	high non-clonal	1	2.06	1	8	0.383224409	NaN	0.30389313	0.467715447	2	2	NaN	NaN	NaN	0.447333333	NaN	No Truncal Mutation detected
RE4F1	RE4	P02	LUNG	called	0.39	3.83	0.55	36	0.183591928	0.191490909	0.163944444	0.1565	2	2	0.675130617	0.076	62.27795193	0.419666667	50.49111808	---
RE4F2	RE4	P02	LUNG	called	0.5	3.81	0.65	29	0.205012195	0.226848485	0.145166667	0.177076923	2	2	0.688477366	0.091	68.24915825	0.2135	57.35129068	---
RE4F3	RE4	P02	LUNG	called	0.22	5.43	0.43	68	0.176941909	0.192266667	0.123045455	0.17203125	1	3	0.680582137	0.089	68.12599681	0.20575	55.2830941	---
RE4F4	RE4	P02	LUNG	called	0.42	3.85	0.58	34	0.180034043	0.194939394	0.1438125	0.145815789	2	2	0.714545455	0.096	67.70562771	0.5995	49.89177489	---
RE6F1	RE6	P03	LUNG	called	0.2	4.03	0.33	66	0.106431818	0.156	0.085482759	0.1505	0	2	0.659375	0.364	50	0.173	68.75	Only 2 Truncal Mutations detected
RE6F2	RE6	P03	LUNG	called	0.16	1.96	0.16	66	0.106794118	0.12125	0.102037037	0.130333333	0	3	0.575	0.5	50	0.1345	63.33333333	Only 2 Truncal Mutations detected
RE6F3	RE6	P03	LUNG	called	0.23	2.12	0.24	47	0.105585366	0.236	0.0839	0.124	0	2	0.692567568	0.524	62.5	0.327333333	73.64864865	Only 2 Truncal Mutations detected
RE6F4	RE6	P03	LUNG	called	0.16	1.07	0.09	61	0.106481481	0.21825	0.079666667	0.191375	1	2	0.7525	0.52	50	0.072	60.5	Only 2 Truncal Mutations detected
RE8F1	RE8	P04	LUNG	called	0.32	4.21	0.49	46	0.222009821	0.225307027	0.210836364	0.204115108	2	3	0.589563247	0.052	50.40747256	0.296918919	53.80586048	---
RE8F2	RE8	P04	LUNG	high non-clonal	0.58	4.34	0.75	28	0.29151633	0.316179459	0.201833333	0.234288194	2	3	0.655537557	0.084	55.15448764	0.565733333	54.26535877	---
RE8F3	RE8	P04	LUNG	high entropy	0.46	4.39	0.65	35	0.255151779	0.275265946	0.156716418	0.210709559	2	3	0.658003466	0.083	53.60034914	0.359983051	57.10528998	---
RE8F4	RE8	P04	LUNG	called	0.27	3.33	0.38	47	0.192184165	0.198313514	0.113677419	0.187166667	3	4	0.619140554	0.049	50.58159754	0.332363636	51.06664348	---
RE9F1	RE9	P05	LUNG	called	0.22	3.87	0.36	58	0.152	0.408	0.117938776	0.157363095	1	3	NaN	NaN	NaN	0.117214286	NaN	No Truncal Mutation detected
RE9F2	RE9	P05	LUNG	called	0.31	5.63	0.56	55	0.229921642	0.414333333	0.141388235	0.268655556	2	3	NaN	NaN	NaN	0.416	NaN	No Truncal Mutation detected
RE9F3	RE9	P05	LUNG	called	0.25	3.94	0.4	53	0.12454386	0.407	0.111265306	0.0852	2	2	NaN	NaN	NaN	0.08	NaN	No Truncal Mutation detected
RE9F4	RE9	P05	LUNG	called	0.3	4.85	0.51	51	0.156137339	0.408333333	0.112022222	0.162778378	1	2	NaN	NaN	NaN	0.1255	NaN	No Truncal Mutation detected
RE10F1	RE10	P06	LUNG	called	0.35	6.42	0.63	55	0.233589474	0.420242424	0.106076923	0.2807	2	4	0.9335	0.2235	89.71	0.4315	71.11	---
RE10F2	RE10	P06	LUNG	called	0.24	3.98	0.38	56	0.154539216	0.229878788	0.117761905	0.126333333	2	2	0.8304787	0.167	80.57	0.193142857	72.99	---
RE10F3	RE10	P06	LUNG	called	0.26	5.3	0.48	60	0.203761905	0.402121212	0.103064516	0.1735	2	2	0.962121212	0.311	88.13131313	0.336714286	82.07070707	---
RE10F4	RE10	P06	LUNG	called	0.35	4.34	0.54	43	0.203847826	0.376272727	0.08216	0.247666667	2	2	0.942218798	0.273	91.88495121	0.332857143	76.91319979	---
RE5F1	RE5	P07	OV	non-aneuploid	NaN	NaN	NaN	NaN	0.341515366	0.456792381	0.119782946	0.288920635	4	3	0.953009939	0.278	93.77748109	0.547	55.3600356	---
RE5F2	RE5	P07	OV	non-aneuploid	NaN	NaN	NaN	NaN	0.351859281	0.385497143	0.176089109	0.354071429	2	3	0.767505828	0.188	76.35164835	0.364184211	67.12687313	---
RE5F3	RE5	P07	OV	non-aneuploid	NaN	NaN	NaN	NaN	0.372459689	0.42759619	0.138438095	0.315649351	1	3	0.858644689	0.258	82.34065934	0.378807692	79.35531136	---
RE5F4	RE5	P07	OV	non-aneuploid	NaN	NaN	NaN	NaN	0.391212257	0.431544762	0.17428125	0.3839375	2	6	0.81843254	0.28	78.18055556	0.424096774	68.52380952	---
RE11F1	RE11	P08	OV	called	0.27	3.6	0.4	48	0.182677632	0.214707071	0.109380952	0.174272727	2	3	0.825995807	0.093	80.05526968	0.458	53.09700781	---
RE11F2	RE11	P08	OV	called	0.25	3.02	0.34	48	0.22096988	0.273212121	0.119104167	0.206105263	4	3	0.80830695	0.122	79.05924921	0.383	57.59837178	---
RE11F3	RE11	P08	OV	called	0.25	5.06	0.46	59	0.219164894	0.279626263	0.135373134	0.202272727	1	3	0.806037907	0.123	79.04891613	0.15175	77.1422086	---
RE11F4	RE11	P08	OV	called	0.22	4.74	0.4	65	0.225337079	0.294050505	0.1038	0.220416667	1	3	0.837424882	0.152	80.39253292	0.2655	67.40825981	---
RE13F1	RE13	P09	OV	called	0.47	5.47	0.71	41	0.196666667	0.242578125	0.126136364	0.215	2	2	0.783166274	0.138	78.19870283	0.370333333	55.76356132	---
RE13F2	RE13	P09	OV	called	0.32	5.19	0.55	50	0.254631944	0.317359375	0.103151515	0.275574468	1	3	0.741113281	0.229	71.875	0.259411765	66.40625	---
RE13F3	RE13	P09	OV	called	0.31	5.3	0.55	52	0.267333333	0.34478125	0.112880952	0.299893617	1	3	0.738851826	0.172	72.05933989	0.222090909	64.9315309	---
RE13F4	RE13	P09	OV	called	0.33	5.21	0.57	49	0.295875969	0.355515625	0.172761905	0.267886364	1	2	0.705288462	0.145	69.19471154	0.229583333	64.42307692	---
RE7F1	RE7	P10	BRCA	called	0.82	2.3	0.84	11	0.31790099	0.44826087	0.1773125	0.425285714	1	2	0.832806324	0.289	82.74703557	0.325	79.48616601	---
RE7F2	RE7	P10	BRCA	called	0.78	2.32	0.81	12	0.3082	0.423347826	0.131810811	0.483857143	1	2	0.863142292	0.304	86.56126482	0.2225	83.05335968	---
RE7F3	RE7	P10	BRCA	called	0.29	5.98	0.55	59	0.311932584	0.433630435	0.11159375	0.385818182	1	2	0.861223458	0.3	89.76238625	0.269	86.27401416	---
RE7F4	RE7	P10	BRCA	called	0.22	3.61	0.33	59	0.19171134	0.237978261	0.143681818	0.189571429	1	2	0.805626598	0.132	83.01364024	0.113666667	81.15942029	---
RE12F1	RE12	P11	UCEC	high non-clonal	0.63	2.24	0.66	14	0.221503448	* 0.323226666666667	0.112514286	NaN	2	2	0.903142857	0.164	88.28571429	0.31075	73.85714286	---
RE12F4	RE12	P11	UCEC	called	0.66	4.45	0.81	26	0.267685185	* 0.4072	0.14737931	NaN	3	3	0.863984674	0.194	82.59770115	0.9165	52.09195402	---
RE14F1	RE14	P12	UCEC	called	0.2	4.62	0.37	68	0.217402299	0.27652	0.110096774	0.279166667	4	3	0.885135135	0.121	86.83783784	0.518	52.2972973	---
RE14F2	RE14	P12	UCEC	called	0.26	5.79	0.51	62	0.254321839	0.30418	0.1312	0.303083333	2	3	0.817567568	0.178	82.83783784	0.5225	51.94594595	---
RE14F3	RE14	P12	UCEC	called	0.23	4.9	0.43	62	0.295	0.4168	0.101848485	0.3215	1	3	0.882173913	0.174	85.04347826	0.4595	60.65217391	---
RE14F4	RE14	P12	UCEC	called	0.22	5.48	0.44	68	0.295521277	0.423	0.110857143	0.305444444	1	3	0.888636364	0.197	88.90909091	0.495	63.72727273	---
RE15F1	RE15	P13	LIHC	called	0.43	4.05	0.61	35	0.266176	0.35	0.225596774	0.304672131	1	3	0.62601626	0.75	50	0.2527	46.54471545	Only 1 Truncal Mutation detected
RE15F2	RE15	P13	LIHC	called	0.23	1.96	0.23	46	0.179296296	0.2475	0.176842105	0.164333333	0	4	0.72	0.667	50	0.428666667	73	Only 1 Truncal Mutation detected
RE15F3	RE15	P13	LIHC	called	0.23	2.04	0.23	47	0.166075949	0.3635	0.15	0.163171875	0	0	0.948051948	1	50	NaN	NaN	Only 1 Truncal Mutation detected
RE15F4	RE15	P13	LIHC	called	0.2	3.91	0.32	66	0.1189	0.28	0.084888889	0.1479	0	0	0.946428571	0.667	50	NaN	NaN	Only 1 Truncal Mutation detected

* data analysed based on two sample biopsies instead of four.
