# Supplementary material for: Whole Exome Sequencing of Multi-Regional Biopsies from Metastatic Lesions to Evaluate Actionable Truncal Mutations Using a Single-Pass Percutaneous Technique
Source: Cancers (Basel). 2020 Jun 17;12(6):1599. doi: 10.3390/cancers12061599 (PMC7353029; doi:10.3390/cancers12061599)
Supplement: Supplementary file 1 [file cancers-12-01599-s001.pdf]

Whole Exome Sequencing of Multi-Regional Biopsies from Metastatic Lesions to Evaluate Actionable Truncal Mutations Using a Single-Pass Percutaneous Technique

Valerie Heong, Darwin Tay, Shane Ee Goh, Bernard Wee, Tuan Zea Tan, Ross Soo, Brendan Pang, Diana Lim, Anil Gopinathan, Samuel Ow, Cheng Ean Chee, Boon Cher Goh, Soo Chin Lee, Wei Peng Yong, Andrea Wong, Mohamed Feroz Mohd Omar, Richie Soong and David SP Tan

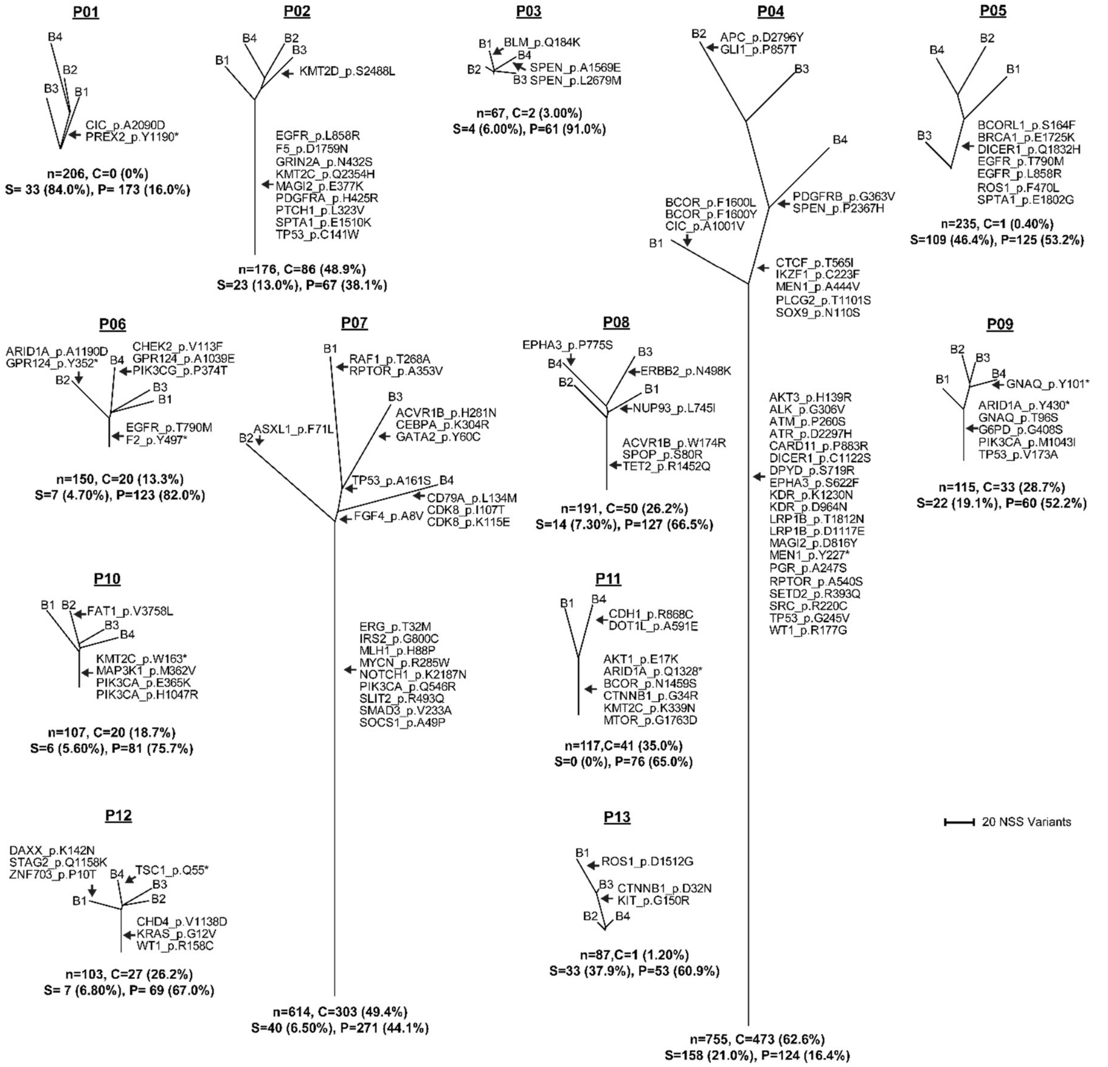

Figure S1. Phylogenetic trees constructed from NSS mutations. The length of each line representing the phylogenetic trees is proportional to the number of NSS variants. The mutated genes and proteins (that reflect genes from FoundationOne™ cancer gene panel) are annotated next to the phylogenetic tree. 'Bx' signifies MRTB sample with identification number x. The total number of NSS, truncal (percentage), branch (percentage), and private (percentage) mutations are denoted by 'n', 'C', 'S', and 'P' respectively. Truncal, branch, and private refer to the number of variants that occur in all, in some but not all, and only one MRTB sample(s) resected from the patient respectively.

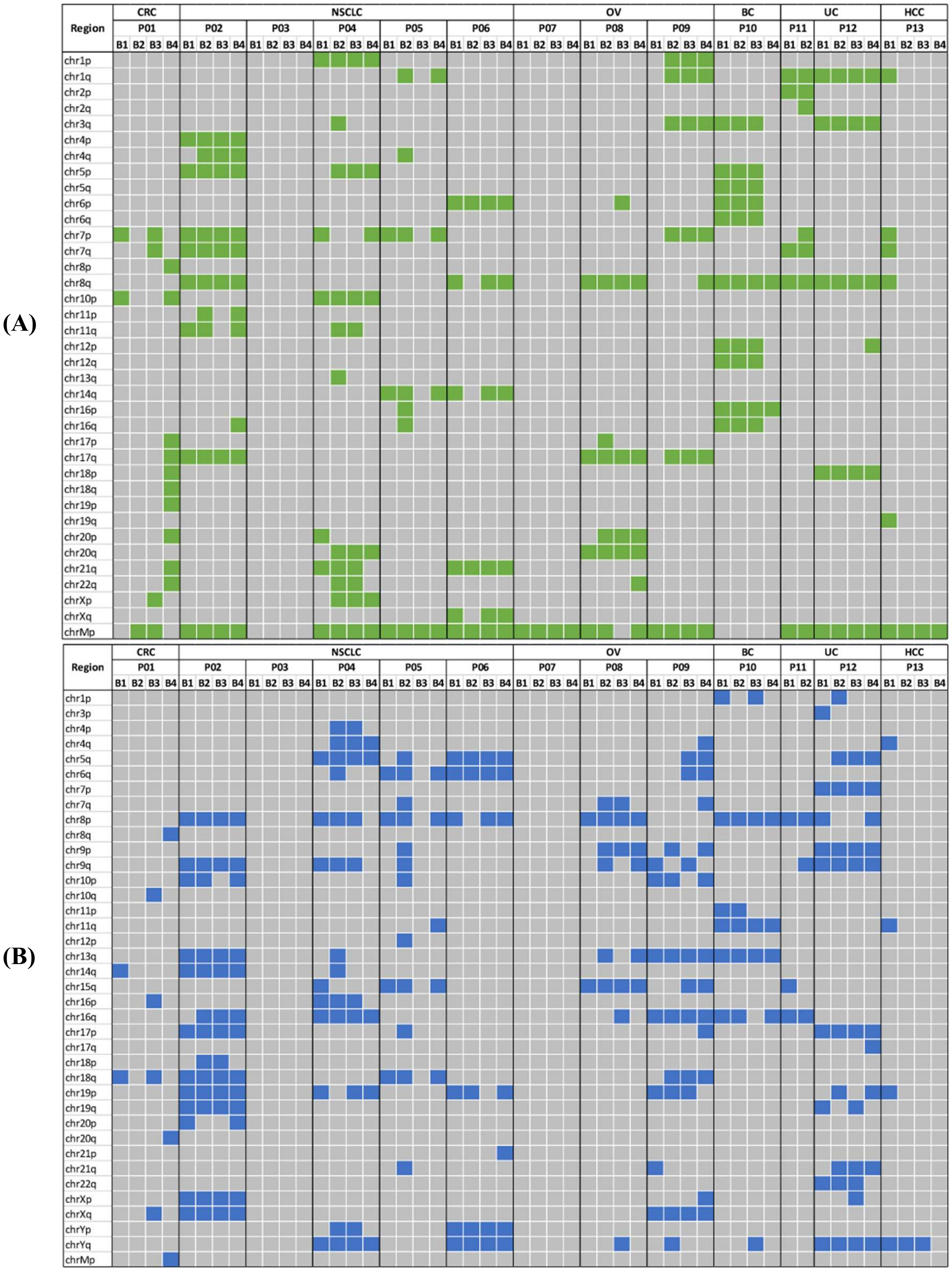

Figure S2. Heatmap visualization illustrating the presence/absence of copy number alterations in relation to each region. (**A**) Green and gray areas represent the presence and absence of large-scale amplification respectively. (**B**) Blue and gray area denote the presence and absence of large-scale deletion respectively. 'Bx' signifies MRTB sample with identification number x.

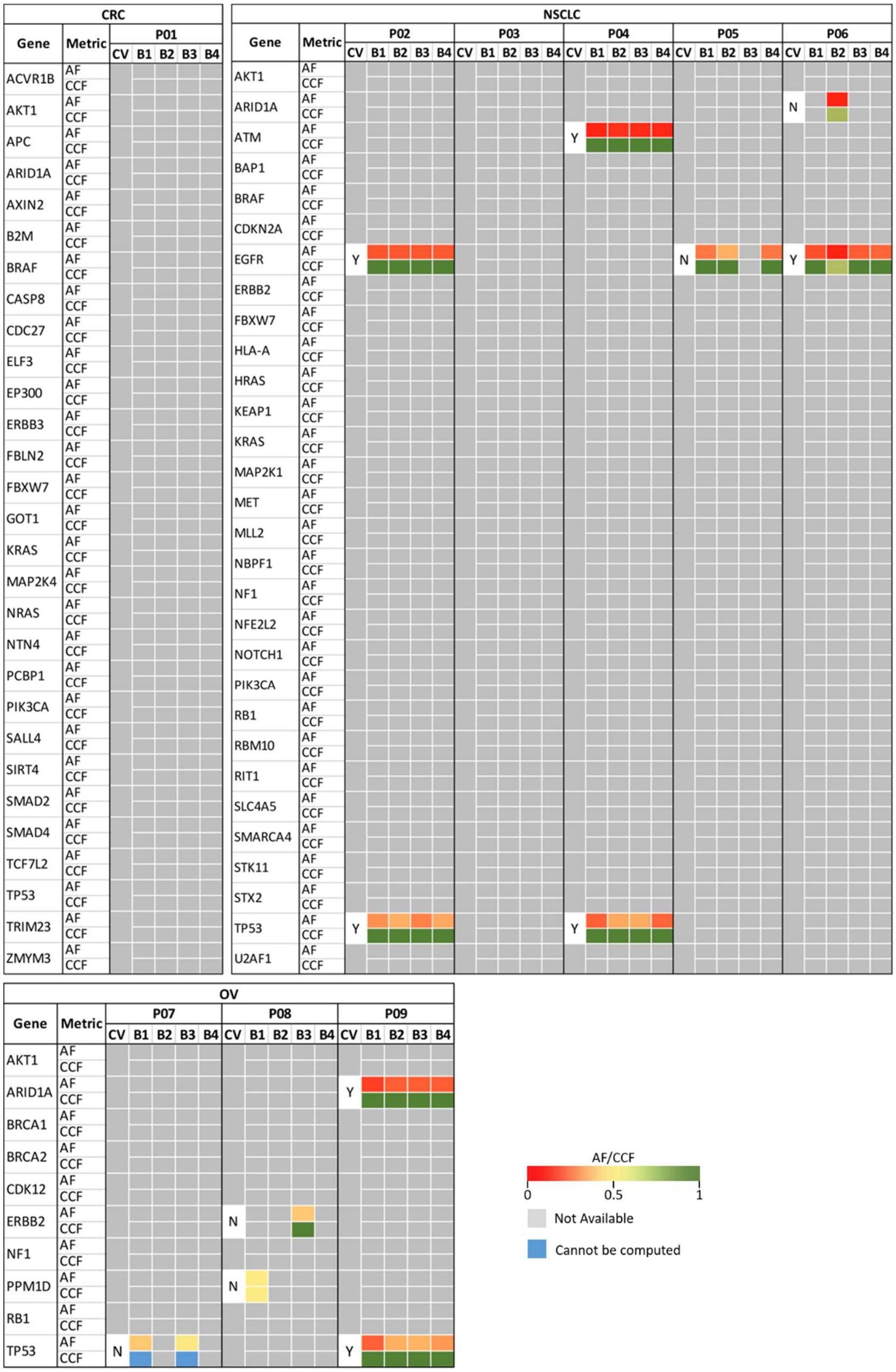

Figure S3. ssCDMs for CRC, NSCLC, and OV. 'CV' refers to whether the variant is truncal (or branch). 'Y' indicates 'yes' while 'N' denotes 'no'. 'AF' represents allele frequency and 'CCF' means cancer cell fraction. 'Bx' signifies MRTB sample with identification number x.

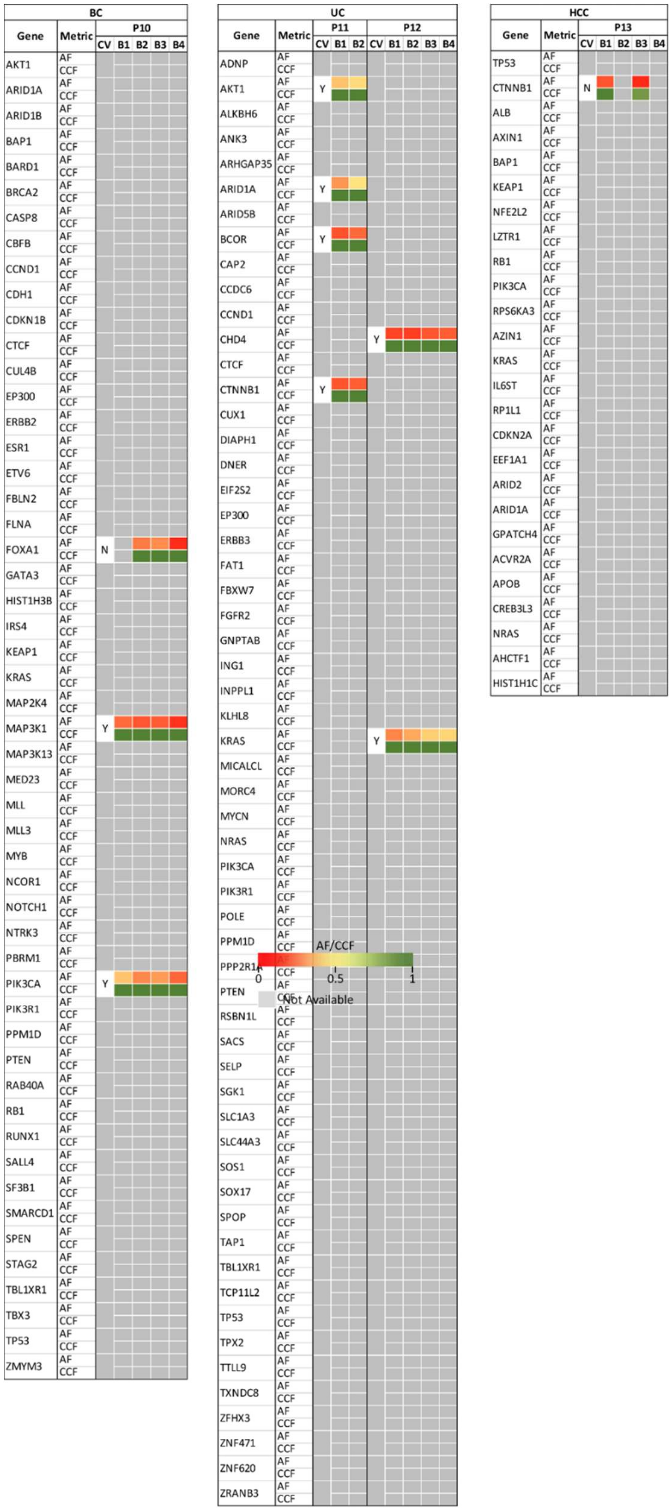

Figure S4. ssCDMs for BC, UC, and HCC. 'CV' refers to whether the variant is truncal (or branch). 'Y' indicates 'yes' while 'N' denotes 'no'. 'AF' represents allele frequency and 'CCF' means cancer cell fraction. 'Bx' signifies MRTB sample with identification number x.

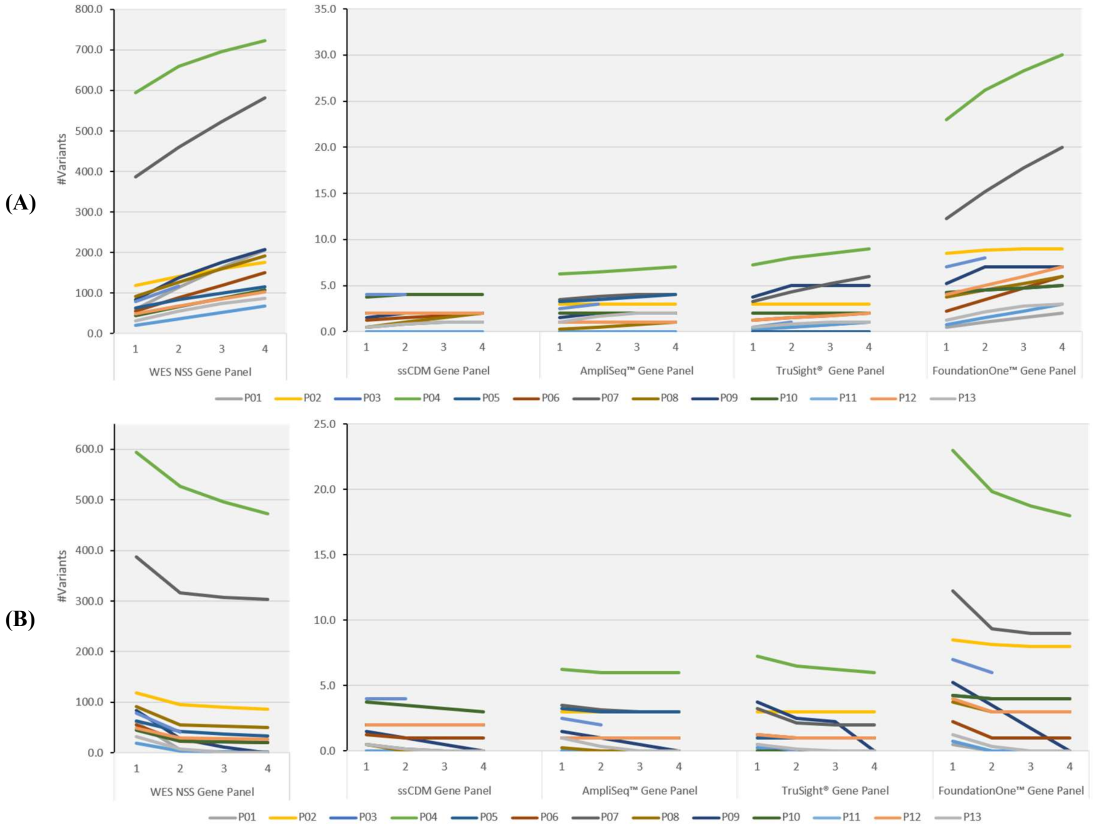

Figure S5. Mutational characteristics across different number of MRTB samples and gene panels. (**A**) Average number of unique variants when different number of MRTB samples were analyzed simultaneously. (**B**) Average number of PTVs detected when different number of MRTB samples were analyzed concurrently. Five gene panels were scrutinized – namely WES NSS, ssCDM, AmpliSeq™, TruSight® and FoundationOne™ cancer gene panels comprising of whole-exome, statistically significant cancer-specific genes, 50, 94, and 315 genes respectively.

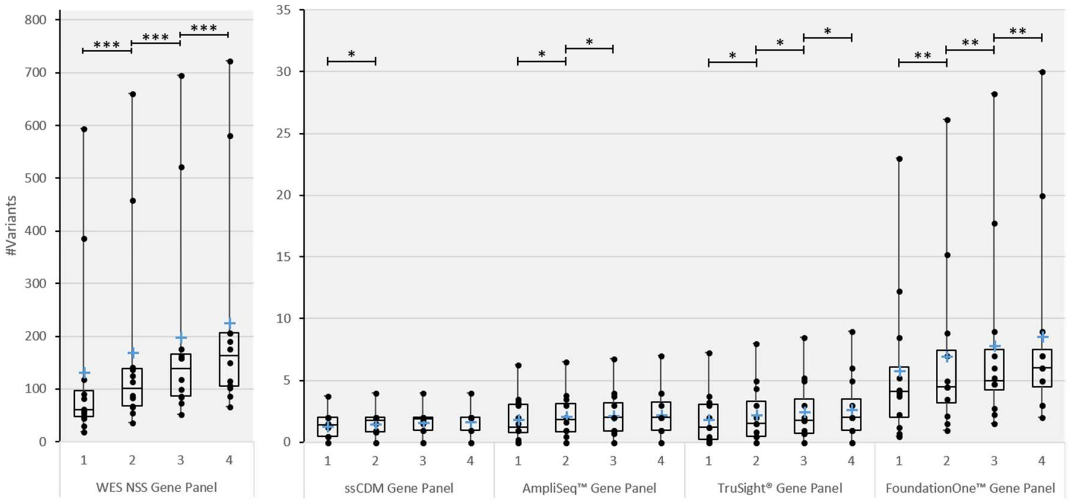

Figure S6. Boxplot illustrating the average number of unique variants across different gene panels and number of MRTB samples. Single asterisk (*) denotes $p < 0.05$, double asterisks (**) signify $p < 0.01$ while triple asterisks (***) indicate $p < 0.001$. Cross (+) represents the mean value of the data.

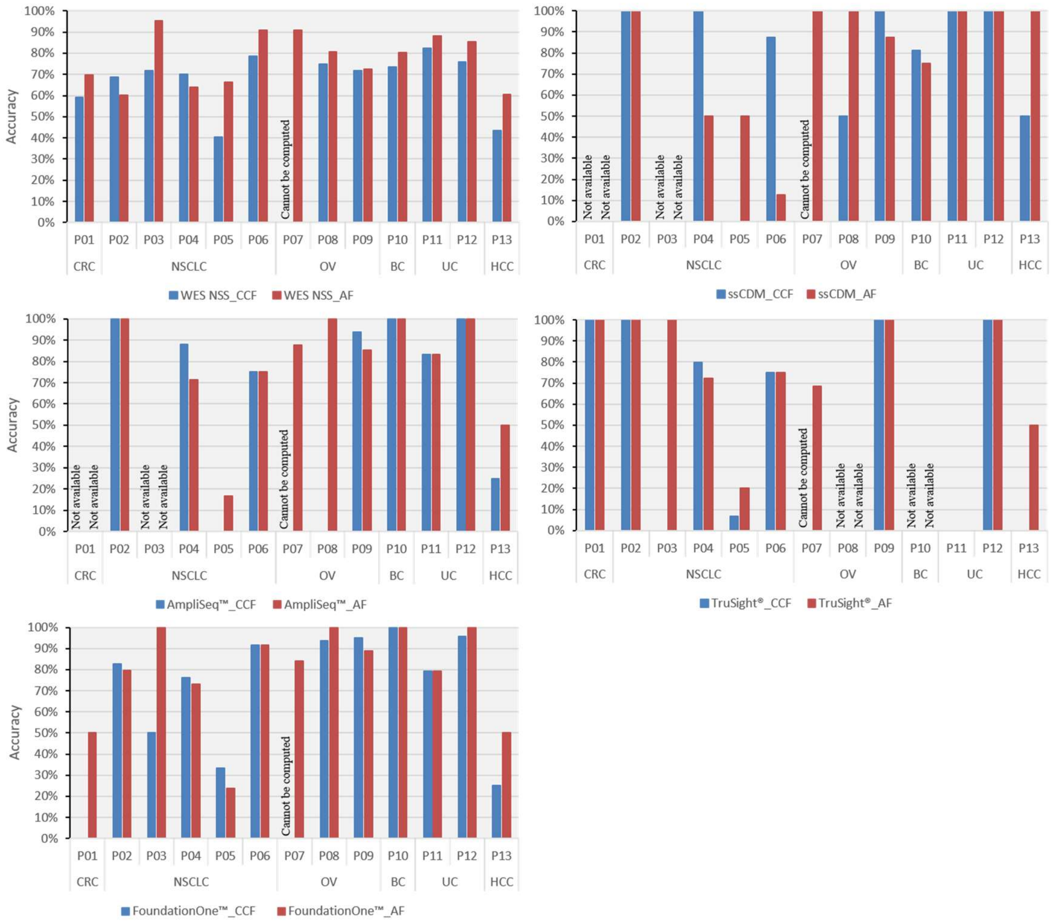

Figure S7. Best average prediction accuracy of PTVs across different patients. Two types of threshold were used to classify variants into either truncal or branch – namely allele frequency (AF) and cancer cell fraction (CCF). Based on the respective threshold, the best average prediction accuracy achievable (within the defined search domain) among all patient (across different gene panels) is portrayed above. 'Not available' signifies that no variant that is associated with the specific gene panel was found. 'Cannot be computed' indicates that the CCF values cannot be estimated because of inadequate information related to somatic copy number alteration.

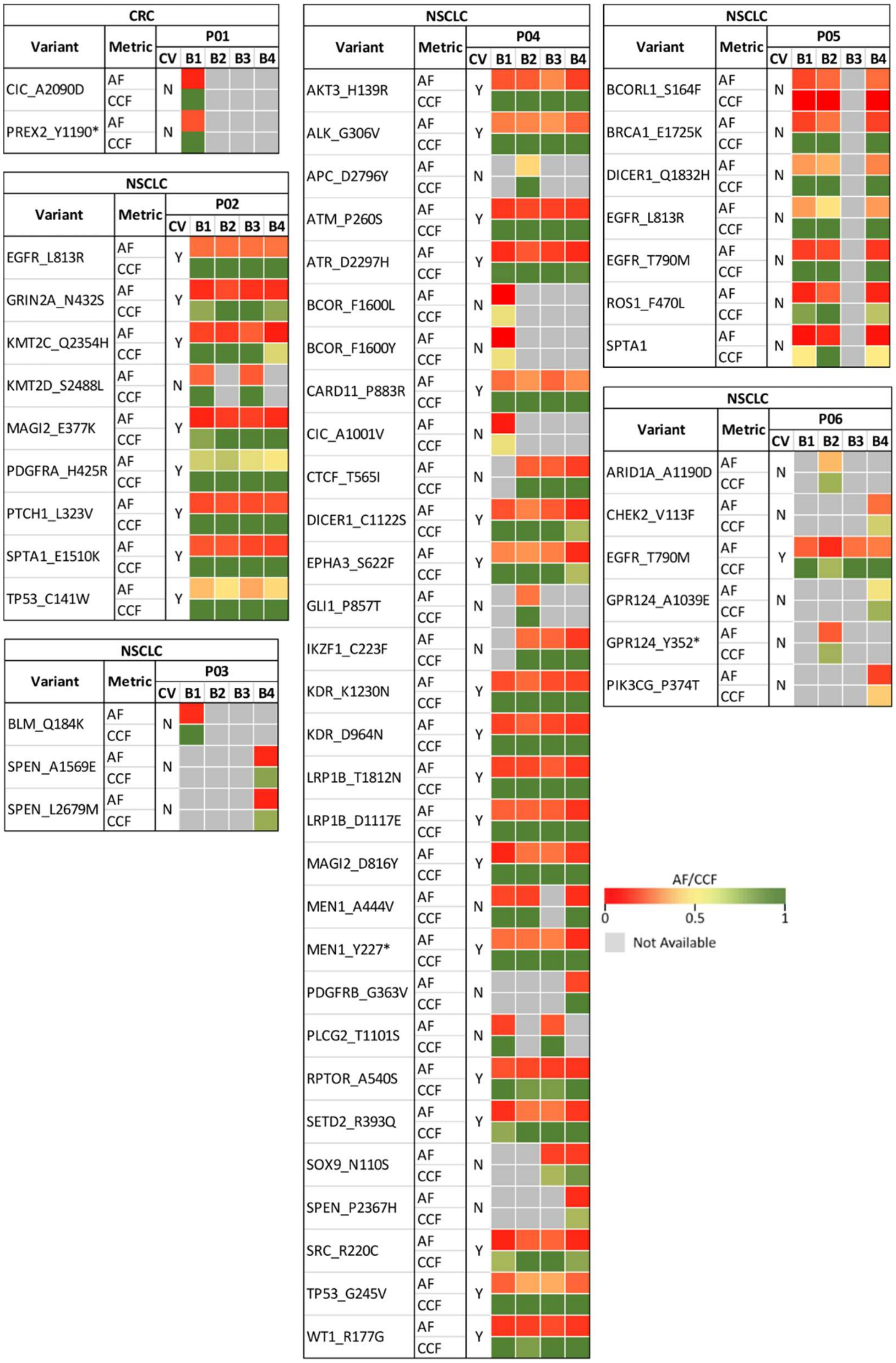

Figure S8. Illustrative example of patients P01-P06 mutational profile. The mutational landscape of each patient that reflects the genes from FoundationOne™ gene panel is shown above. 'CV' refers to whether the variant is truncal (or branch). 'Y' indicates 'yes' for truncal variant while 'N' denotes 'no' indicating a branch variant. 'AF' represents allele frequency and 'CCF' means cancer cell fraction. 'Bx' signifies MRTB sample with identification number x.

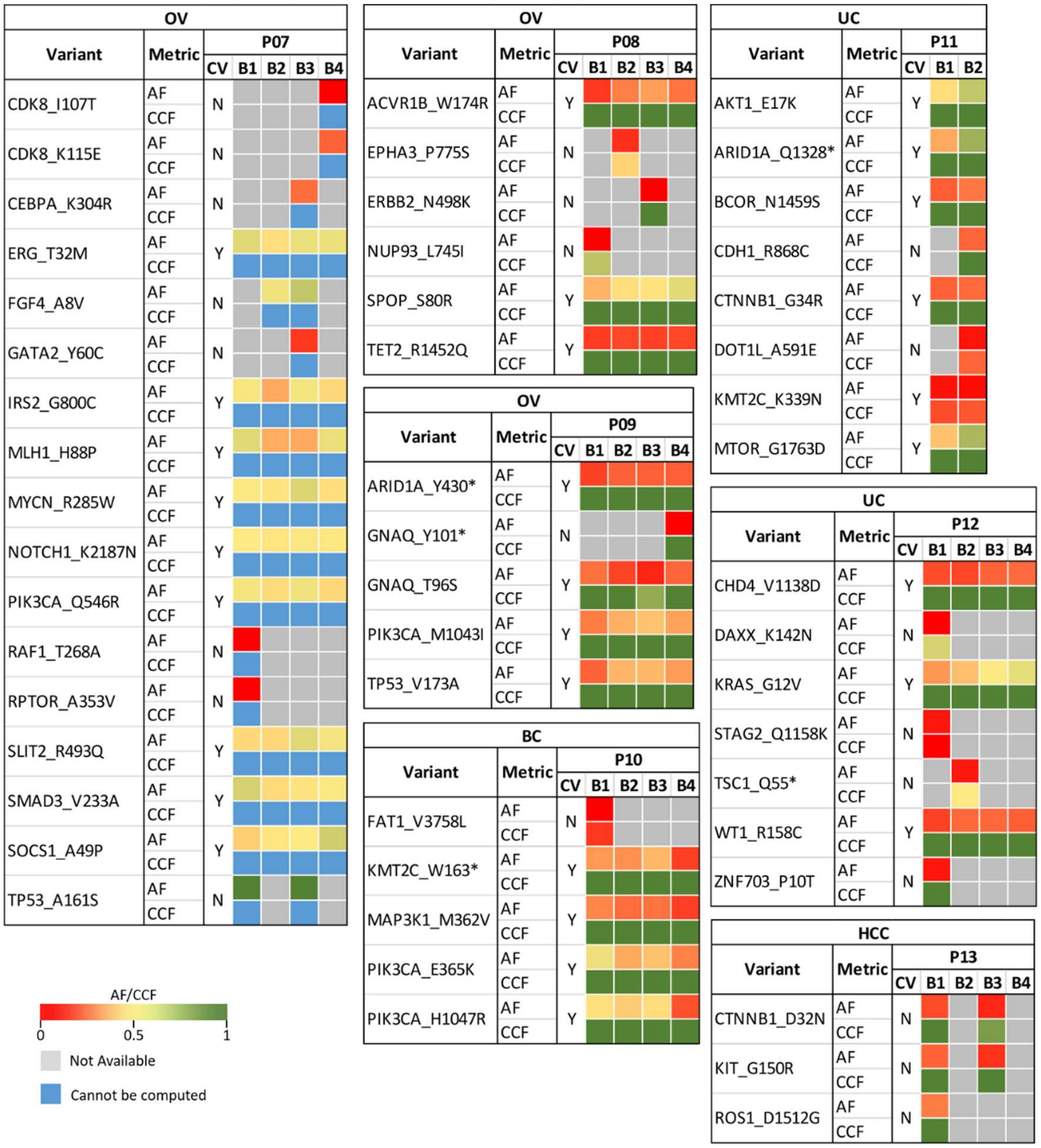

Figure S9. Illustrative example of patients P07-P13 mutational profile. The mutational landscape of each patient that reflects the genes from FoundationOne™ gene panel is shown above. 'CV' refers to whether the variant is truncal (or branch). 'Y' indicates 'yes' for truncal variant while 'N' denotes 'no' indicating a branch variant. 'AF' represents allele frequency and 'CCF' means cancer cell fraction. 'Bx' signifies MRTB sample with identification number x.

Chrom	Pos	Ref	Alt	cDNA_Change	Protein_Change	Symbol	RE10F1	RE10F2	RE10F3	RE10F4
22	2.9E+07	C	A	c.337G>T	p.V113F	CHEK2				
8	3.8E+07	C	A	c.3116C>A	p.A1039E	GPR124				
7	1.1E+08	C	A	c.1120C>A	p.P374T	PIK3CG				
1	2.7E+07	C	A	c.3569C>A	p.A1190D	ARID1A				
8	3.8E+07	C	A	c.1056C>A	p.Y352*	GPR124				
7	5.5E+07	C	T	c.2234C>T	p.T790M	EGFR				

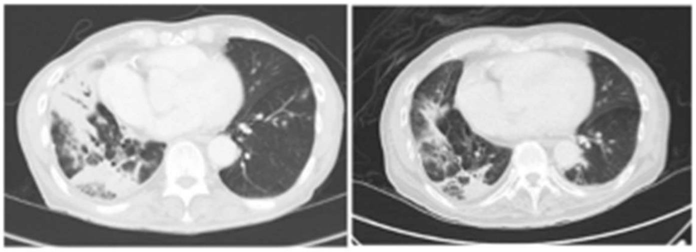

Chrom	Pos	Ref	Alt	cDNA_Change	Protein_Change	Symbol	RE12F1	RE12F4
16	6.9E+07	C	T	c.2602C>T	p.R868C	CDH1		
19	2213960	C	A	c.1772C>A	p.A591E	DOT1L		
14	1.1E+08	C	T	c.49G>A	p.E17K	AKT1		
1	2.7E+07	C	T	c.3982C>T	p.Q1328*	ARID1A		
X	4E+07	T	C	c.4376A>G	p.N1459S	BCOR		
3	4.1E+07	G	A	c.100G>A	p.G34R	CTNNB1		
7	1.5E+08	C	G	c.1017G>C	p.K339N	KMT2C		
1	1.1E+07	C	T	c.5288G>A	p.G1763D	MTOR		

Aug 2015 Oct 2015

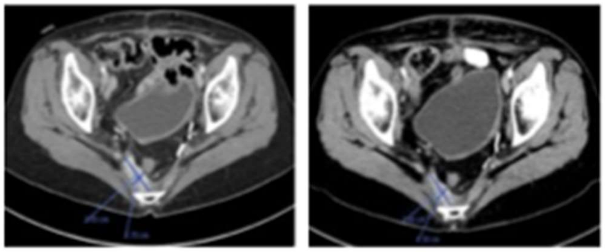

Figure S10. Pre - treatment and 8- week post treatment images from. (**A** – panel above) Patient P06 who was treated with a EGFR T790M inhibitor and (**B**- panel below) Patient P11 who was treated with a pan-AKT inhibitor. Pre-treatment and 8-week post treatment scans were not available for patients P5 and P10 as their disease progressed clinically prior to repeat imaging scans.

Table S1. Average number of unique variants detected across different number of MRTB samples and gene panels.

Panel	Samples	CRC	NSCLC						OC		BC	UC		HCC
		P01	P02	P03	P04	P05	P06	P07	P08	P09	P10	P11	P12	P13
WES NSS	1	60.0	118.0	19.5	593.8	83.0	55.8	387.3	90.8	62.8	44.5	79.0	48.8	31.3
	2	114.2	141.2	36.0	660.0	138.0	88.3	458.8	126.5	83.8	66.3	117.0	68.0	55.3
	3	162.8	159.3	51.8	695.3	175.8	119.3	522.3	159.3	100.0	86.8	NA	85.8	73.8
	4	206.0	176.0	67.0	723.0	207.0	150.0	582.0	191.0	115.0	107.0	NA	103.0	87.0
ssCDM	1	0.0	2.0	0.0	2.0	1.5	1.3	0.5	0.5	2.0	3.8	4.0	2.0	0.5
	2	0.0	2.0	0.0	2.0	2.0	1.5	0.8	1.0	2.0	4.0	4.0	2.0	0.8
	3	0.0	2.0	0.0	2.0	2.0	1.8	1.0	1.5	2.0	4.0	NA	2.0	1.0
	4	0.0	2.0	0.0	2.0	2.0	2.0	1.0	2.0	2.0	4.0	NA	2.0	1.0
AmpliSeq™	1	0.0	3.0	0.0	6.3	1.5	1.0	3.5	0.3	3.3	2.0	2.5	1.0	1.0
	2	0.0	3.0	0.0	6.5	2.0	1.0	3.8	0.5	3.5	2.0	3.0	1.0	1.7
	3	0.0	3.0	0.0	6.8	2.0	1.0	4.0	0.8	3.8	2.0	NA	1.0	2.0
	4	0.0	3.0	0.0	7.0	2.0	1.0	4.0	1.0	4.0	2.0	NA	1.0	2.0
TruSught®	1	0.3	3.0	0.3	7.3	3.8	1.3	3.3	0.0	0.0	2.0	0.5	1.3	0.5
	2	0.5	3.0	0.5	8.0	5.0	1.5	4.3	0.0	0.0	2.0	1.0	1.5	0.8
	3	0.8	3.0	0.8	8.5	5.0	1.8	5.3	0.0	0.0	2.0	NA	1.8	1.0
	4	1.0	3.0	1.0	9.0	5.0	2.0	6.0	0.0	0.0	2.0	NA	2.0	1.0
FoundationOne™	1	0.5	8.5	0.8	23.0	5.3	2.3	12.3	3.8	4.3	4.3	7.0	4.0	1.3
	2	1.0	8.8	1.5	26.2	7.0	3.5	15.2	4.5	4.5	4.5	8.0	5.0	2.2
	3	1.5	9.0	2.3	28.3	7.0	4.8	17.8	5.3	4.8	4.8	NA	6.0	2.8
	4	2.0	9.0	3.0	30.0	7.0	6.0	20.0	6.0	5.0	5.0	NA	7.0	3.0

Unique variant refers to any variant that appears in at least one of the MRTB samples analyzed simultaneously. 'NA' denotes not available. NSCLC = Non-small cell lung carcinoma, CRC = colorectal carcinoma, OC = ovarian carcinoma, BC = breast carcinoma, UC = uterine carcinoma and HCC = hepatocellular carcinoma.

Table S2. Average number of PTVs detected across different number of MRTB samples and gene panels.

Panel	Samples	CRC	NSCLC						OV		BC	UC		HCC
		**P01**	**P02**	**P03**	**P04**	**P05**	**P06**	**P07**	**P08**	**P09**	**P10**	**P11**	**P12**	**P13**
WES NSS	1	60.0	118.0	19.5	593.8	83.0	55.8	387.3	90.8	62.8	44.5	79.0	48.8	31.3
	2	5.8	94.8	3.0	527.5	28.0	23.2	315.7	55.0	41.7	22.7	41.0	29.5	7.2
	3	0.3	89.8	2.3	496.5	10.8	21.5	307.5	52.0	36.8	21.3	NA	28.0	1.5
	4	0.0	86.0	2.0	473.0	0.0	20.0	303.0	50.0	33.0	20.0	NA	27.0	1.0
ssCDM	1	0.0	2.0	0.0	2.0	1.5	1.3	0.5	0.5	2.0	3.8	4.0	2.0	0.5
	2	0.0	2.0	0.0	2.0	1.0	1.0	0.2	0.0	2.0	3.5	4.0	2.0	0.2
	3	0.0	2.0	0.0	2.0	0.5	1.0	0.0	0.0	2.0	3.3	NA	2.0	0.0
	4	0.0	2.0	0.0	2.0	0.0	1.0	0.0	0.0	2.0	3.0	NA	2.0	0.0
AmpliSeq™	1	0.0	3.0	0.0	6.3	1.5	1.0	3.5	0.3	3.3	1.0	2.5	1.0	1.0
	2	0.0	3.0	0.0	6.0	1.0	1.0	3.2	0.0	3.0	1.0	2.0	1.0	0.3
	3	0.0	3.0	0.0	6.0	0.5	1.0	3.0	0.0	3.0	1.0	NA	1.0	0.0
	4	0.0	3.0	0.0	6.0	0.0	1.0	3.0	0.0	3.0	1.0	NA	1.0	0.0
TruSught®	1	0.3	3.0	0.3	7.3	3.8	1.3	3.3	0.0	1.0	0.0	0.5	1.3	0.5
	2	0.0	3.0	0.0	6.5	2.5	1.0	2.2	0.0	1.0	0.0	0.0	1.0	0.2
	3	0.0	3.0	0.0	6.3	2.3	1.0	2.0	0.0	1.0	0.0	NA	1.0	0.0
	4	0.0	3.0	0.0	6.0	0.0	1.0	2.0	0.0	1.0	0.0	NA	1.0	0.0
FoundationOne™	1	0.5	8.5	0.8	23.0	5.3	2.3	12.3	3.8	4.3	4.3	7.0	4.0	1.3
	2	0.0	8.2	0.0	19.8	3.5	1.0	9.3	3.0	4.0	4.0	6.0	3.0	0.3
	3	0.0	8.0	0.0	18.8	1.8	1.0	9.0	3.0	4.0	4.0	NA	3.0	0.0
	4	0.0	8.0	0.0	18.0	0.0	1.0	9.0	3.0	4.0	4.0	NA	3.0	0.0

Using the number of MRTB samples analyzed simultaneously as the baseline reference, the average number of putative truncal variants is determined. 'NA' denotes not available.

File S1. Study Protocol.

Study Protocol (REMATCH)

STUDY TITLE:

Rebiopsy for Molecular Analysis of Tumours to Identify Clonal Actionability, Evolution and Heterogeneity (REMATCH)

INSTITUTION:

National University Hospital (NUH), National University Cancer Institute (NCIS) and Cancer Science Institute (CSI), Singapore

PRINCIPAL INVESTIGATOR:

Dr David SP Tan

Department of Hematology-Oncology

National University Cancer Institute (NCIS)

National University Health System (NUHS)

1. E Kent Ridge Road

Singapore 119228

David_SP_Tan@nuhs.edu.sg

CO-INVESTIGATORS:

Dr Ross Soo

Dr. Valerie Heong

Dr Anil Gopinathan

Dr Bernard Wee

A/Prof Goh Boon Cher

A/Prof Lee Soo Chin

Dr Yong Wei Peng

A/Prof Richie Soong

Dr Diana GZ Lim

Dr Brendan Pang

Abstract: Malignant tumours are composed of multiple clonal subpopulations of cells, which may have differing karyotypes, growth rates, metastatic potential, immunological characteristics, gene expression profiles and sensitivity to treatment. This intratumoral cellular diversity, or tumour heterogeneity, has a profound impact on diagnosis, disease progression and response to therapy. A single biopsy from a single region at a single time point may provide a small sample of tumor cells sufficient for diagnostic purposes, but may not be representative of the diversity of the entire cancer cell population. In addition, different clones will evolve genetically and progress and respond to treatment in different ways. Despite the importance of tumor heterogeneity and evolution, it is a subject that remains poorly studied. This is primarily due to the challenge of obtaining representative tumour samples. In this study, we will explore the feasibility of obtaining multi-region tumour samples using either interventional radiology techniques or during surgical resection to obtain tumour specimens in order to identify actionable mutations and study the impact of tumour heterogeneity and clonal evolution on treatment outcomes. Patients with advanced cancer and who have biopsiable tumours will be subject to fine needle biopsies (FNBs) and/or core needle biopsies (CNBs) of their tumours while patients undergoing surgical exploration or resection procedures will have selected section/s of tumour from multiple regions biopsied/resected. Depending on the location of the tumour, either a multiple spatial sampling technique will be employed using a coaxial approach to enable different parts of a single tumour to be sampled or a single pass sampling technique will be used during the biopsy procedure. For patients undergoing surgical procedures, multi-region sampling will be obtained from specific sites of tumour. DNA/RNA/protein will then be extracted from the biopsy samples and subject to Next Generation Sequencing, flow cytometry, immunohistochemistry, immune profiling techniques to (1) identify actionable mutations; (2) compare the genetic and immunologic profiles obtained from FNBs and CNBs; (3) evaluate intratumoural heterogeneity by comparing protein, genetic and immunologic profiles of the different areas sampled in each tumour; (4) compare the protein, genetic and immune profiles of the metastatic/ progressive lesions with primary tumours to assess clonal evolution.

1. Background and Rationale

Genetic alterations in cancer are the hallmark of the disease and constitute the most consistent biomarkers predictive of benefit or resistance to targeted cancer treatments [1–9]. Novel molecular sequencing technologies and platforms provide the opportunity to more comprehensively characterize molecular aberrations of human cancers for individual cancer patients. This strategy enables personalization of therapies targeted to the molecular pathology and genomics of individual patients and their malignancies with greater specificity than currently available. In addition, immunophenotypic analysis is becoming increasingly utilized to improve our understanding of the immune microenvironment to aid patient selection and personalization of treatment with immunotherapeutics. Molecular profiling and immunophenotyping of tumors using genomic and immunophenotyping technologies for somatic mutations, gene amplifications and immunomonitoring have allowed the subsequent matching of these results with available cancer therapeutics for cancer patients, are being actively pursued by drug development programs worldwide.

1.1. The Challenge of Tumour Heterogeneity in the Era of Personalised Cancer Care

It has long been recognized that a tumor is composed of multiple clonal subpopulations of malignant cells, which have differing karyotypes, growth rates, metastatic potential, immunological characteristics, gene expression profiles and sensitivity to treatment [10]. This intratumoral cellular diversity, or tumor heterogeneity, has a profound impact on diagnosis, disease progression and response to therapy [11]. A single biopsy from a single region at a single time point may only provide a small sample of tumor cells, which may not be representative of the diversity of the entire cancer cell population. In addition, different clones will progress and respond to treatment in different ways. Despite the obvious importance of tumor heterogeneity, it is a subject that remains poorly explored to date due to several issues [12]. As mentioned above, obtaining a representative sample of the tumor

is an obvious challenge, but sample size can also be a problem [13]. If the tumor sample is too large, for instance in the case of a surgical specimen, then the signal from smaller pools of clones will be lost as "noise". Thus an average of the population will be detected which typically reflects only the dominant clone(s). Deep sequencing of whole genomes, however, can detect mutant alleles in a small fraction of cells [14]. While analyzing single cells is possible [15] it does not take into account intercellular variability. Ideally analyzing large numbers of individual cancer cells would address the problem of noise and signal, but such an effort has been considered impractical. Studying tumour samples from multi - region spatially distinct sites that represent different morphological units of the entire tumor may be one method to address the noise to signal balance [12]. Another approach to examine tumor heterogeneity would be to limit the focus to a few genetic loci known to be important in tumorigenesis [16]. Again the problem with the limited approach is that important differences may be missed and heterogeneity underestimated. Nonetheless, the advantage of targeted sequencing of select regions of genes, or of whole exomes of genes, that are considered drivers of cancer progression, is that any heterogeneity found is likely to be functionally important.

1.2. Small Volume Samples Obtained by Minimally Invasive Procedures to Evaluate Molecular Heterogeneity in Recurrent Cancer

One of the main challenges impeding rapid identification and validation of biomarkers is obtaining clinically relevant tumor specimens [17]. Surgical specimens or archived samples are often selected for molecular characterization of tumors because they provide a source of abundant DNA. Use of archival tissue avoids subjecting the patient to an invasive biopsy procedure, however the archival sample may not be truly reflective of the molecular profile of the recurrent tumour. Additionally, in many cases, core or needle biopsies will be used to collect tumor samples from patients participating in a prospective clinical trial but it is still not clear whether a single core biopsy will provide a true representation of the molecular heterogeneity of the metastatic lesion. To answer this question, we intend to explore the feasibility of doing multi-region sampling of newly diagnosed or recurrent tumours using minimally invasive techniques (for those undergoing percutaneous radiological procedures) or during surgical procedure as part of routine practice to facilitate genomic and immunophenotypic analysis of tumour cells.

If such an approach is successful, minimally invasive sampling techniques at anatomically difficult tumor sites for genomic analysis may be possible, and may also allow sampling at a variety of tumor sites, facilitating the assessment of tumoral heterogeneity. This would mitigate the risks and costs associated with a surgical procedure, open or excisional biopsy in order to obtain tissue, without compromising the quality of genomic analysis. Sampling techniques associated with better safety and fewer complications could encourage clinicians to recommend patients to undergo serial FNB/CNBs at critical time points of clinically relevant tumor sites. A relevant site would include not only the primary, but also new metastases or a disease site progressing on treatment. If the safety of multiple sampling at a single site using minimally invasive techniques can be established, then minimally invasive techniques may also provide additional data on intralesional tumour heterogeneity as well. This spatial and temporal approach to minimally invasive sampling could potentially enhance the understanding of tumor biology and evolution (including an accurate assessment of treatment response and mechanisms of resistance), and facilitate the selection of therapeutic agents that will target the most therapeutically relevant lesions for the patient.

Clearly there is an urgent need to investigate these issues and the first step in this process will be to determine if such a study is feasible in a clinical setting. Evaluating tumour genomic actionability and immunophenotyping, heterogeneity and clonal evolution concurrently with an assessment of small volume tumor samples, are the major objectives of this prospective exploratory analysis. A more tertiary objective is to assess the possibility of quantifying circulating free tumor DNA (cfDNA) through plasma based nucleic acid detection methods in the clinical setting and how the molecular profiles derived from cfDNA correlates with the molecular profiles obtained from tumour biopsies. The one caveat here will be patient safety and willingness to co-operate in this study. At NCIS/NUHS, however, we have already previously demonstrated that patients are willing to

participate in studies that require biopsy samples to be performed, as well as the safety and feasibility of obtaining pre and post treatment biopsies from recurrent tumours in patients on clinical trials (see below).

2. Preliminary Studies/Progress Reports

2.1. Biomarker-driven therapeutic studies at National University Hospital, Cancer Science Institute and Singapore Immunology Network (SIgN)

The Hematology-Oncology Research Group (HORG), in which this study is anchored, has had extensive experience in conducting studies based on tissue collection, storage, annotation, processing and molecular/genetic profiling. Studies along these lines include an active breast cancer neoadjuvant program since 2000 where serial core biopsies are subjected to gene expression profiling (Affymetrix U133 + 2) in order to identify predictive gene signatures.

We have also recently completed a biomarker study to determine the molecular correlates of clinical benefit to regorafenib, a multiple receptor tyrosine kinase inhibitor that has shown clinical benefit in colorectal cancer (CRC). In this study, patients with refractory CRC, who have tumours amenable to biopsy will be subjected to baseline biopsy and blood and plasma-based biomarker assays, followed by repeat biopsy and biomarker analysis following 21 days of treatment at 160 mg daily of regorafenib.

Other studies include the Phase II clinical trial comparing conventional and genotype-based dose adjustment for warfarin, which has laid the foundation for personalised medicine workflows at the NUH and NUS campus. There are also on-going studies to test the feasibility of next-generation sequencing in the diagnosis of both inherited germline and 'actionable' somatic tumour DNA variants in collaboration with local and overseas diagnostic centres and Illumina Inc.

Similarly, in collaboration with SIgN, we will be conducting a study to identify immunophenotypic markers relevant in ovarian cancer that would include following analysis:

2.2. Flow Cytometry

FACS analysis of the major infiltrating immune subtypes using fresh tumors by our collaborators at Singapore Immunology Network will include but not restricted to PD1, CTLA4 and the exhaustion immune markers of LAG3/TIM3 as well as MHC class II profiles.

2.3. Protein Expression of Tumour Tissue

Protein expression using an automated and high-throughput imaging system that allows multiplexed quantitative analysis using the Vectra for basic immune infiltrates

2.4. Serum Analysis

Blood/Serum will be analysed via Luminex by our collaborators at Singapore Immunology Network for cytokine profiles relevant for ovarian cancer and resistance immunotherapeutic strategies.

3. Study Hypothesis And Aims

3.1. Hypothesis

Multi-region sampling of a malignant lesion, using either a radiologically-guided approach or during surgical resection/staging as part of standard of care in patients with newly diagnosed or advanced cancer, is a feasible and safe approach for obtaining information on actionable molecular targets, genetic heterogeneity and the immune microenvironment using next generation sequencing techniques and immunomonitoring platform.

3.2. Primary Objective:

Demonstration of feasibility and optimization of processes and procedures for different methods of sample collection from patients with advanced cancer with tumours progressing on treatment for genomic and immunophenotypic analyses.

3.3. Secondary Objectives:

1. Determine concordance of genomic analyses between archival samples and fresh core needle biopsy (CNB) samples and/or fresh fine needle biopsy (FNB) samples and, where available, surgical biopsy (SB) specimens. Specifically, this includes determining concordance between samples at different time points and concordance between different sites, namely metastases, sites that are progressing and the primary site.
2. Provide comprehensive information on the processes and procedures; and preliminarily evaluate the feasibility of utilizing small volume tumor samples from FNB/CNBs for next generation sequencing technology and immunomonitoring platforms in a clinical setting.
3. Explore the feasibility and provide preliminary information on the processes and procedures for integrating plasma based nucleic acid detection methods of circulating tumor DNA in a clinical setting.
4. Explore the issue of intratumoural heterogeneity by comparing the molecular profiles and the immune microenvironment of the different areas sampled in tumours where multiple spatial sampling was performed
5. Explore the issue of tumour clonal evolution by comparing the molecular profiles of primary and recurrent cancers

4. Eligibility Criteria

4.1. Inclusion Criteria

(1) ≥21 years.
(2) Patients with a histological or cytological diagnosis of newly diagnosed or advanced/metastatic cancer who are potential candidates for a phase I or II or III clinical trial.
(3) At least one biopsiable lesion deemed medically accessible and safe to biopsy or patient who is undergoing surgical resection/staging as part of routine standard of care.
(4)Candidate for one or more phase I or II clinical trials at the time of study enrollment or at a later time point.
(5) Fulfills local institution's laboratory parameters for tumor biopsy.
(6) Willingness and ability of patient to provide signed voluntary informed consent.
(7) Have a performance status of 0–1

4.2. Exclusion Criteria

(1) Any condition that could interfere with a patient's ability to provide informed consent such as dementia or severe cognitive impairment.
(2) Any contraindication to undergoing a biopsy procedure

5. Methods

5.1. Study Design

This multidisciplinary study will investigate the suitability of small volume tumor samples for genomic analysis and immunoprofiling, particularly deep sequencing, and concomitantly it will explore functionally significant tumor heterogeneity and clonal evolution. This study will involve 1 prospective feasibility cohort study, and 1 sub-study of cfDNA, of patients with a confirmed histological or cytological diagnosis of newly diagnosed or advanced recurrent cancer, who are potential candidates for a phase I or II or III clinical trial:

5.2. Study (N = 35)

Multiple sampling of newly diagnosed or recurrent tumours during routine surgical resection/ staging procedures or using a minimally-invasive radiologically guided co-axial technique to identify molecular actionability and heterogeneity and immunophenotyping in cancer by next generation sequencing and immunomonitoring techniques.

To assess whether multiple samples of individual primary/metastatic/recurrent tumours during routine surgical resection/staging procedure or using minimally invasive techniques can provide sufficient material for analysis to identify actionable genetic mutations and data on intratumoural heterogeneity. A maximum of 6 CNBs will be obtained per patient for patients undergoing percutaneous biopsy procedures while a maximum of 10 multi-region biopsies will be obtained per patient for patients undergoing routine surgical resection/staging procedures – one or two cores/samples will be fixed in formalin for diagnostic evaluation and confirmation, and 2–4 others will be fresh frozen and used for next generation sequencing while 4–7 samples will be used for immunophenotyping. Plasma based detection methods for specific circulating tumor DNA fragments will be used to determine the presence and quantity of these fragments. Application of genomic information and immunophenotypic parameters by investigators will be captured. Archived tumor samples with sufficient available tumor tissue, will be requested from all patients for molecular profiling.

5.3. Sub-study cfDNA

The cfDNA obtained from the participants in both studies will be subject to sequencing as well as immunophenotypic analysis in order to identify the presence of any actionable mutations and immune microenvironment identified in the above study.

5.4. Biopsy procedure and risks

Pecutaneous needle biopsy (PNB) is defined as placement of a needle(s) into a suspected abnormal lesion or organ for the purpose of obtaining tissue or cells for diagnosis. Every PNB will be performed using CT scan or ultrasound guidance. The majority of these will be done under local anesthesia and sedation would be employed for select cases depending on the clinical requirement. Fine needle biopsy (FNB) will be done with a 21G or smaller gauged pre-assembled aspiration needle biopsy device. Core needle biopsy (CNB) will involve the use of a 20G or larger spring loaded tru-cut biopsy device**.**

All the biopsies will be performed in the presence of cytotechnician to confirm the representative nature of the site that is being biopsied. Whenever feasible, PNB would be done using a coaxial technique. This entails pre-placement of a guiding needle into the lesion. Subsequently the biopsy needle/device will be coaxially introduced into the lesion through this guiding needle. Using this technique, multiple samples can be obtained through a single pass. It also facilitates sampling of different aspects of the lesion/organ by directing the guiding needle in different directions.

Many variables will affect the eventual success of a PNB procedure. These include the number of samples obtained, the size of the target abnormality, the organ system in which biopsy is performed, the benign or malignant nature of the lesion, the availability of an on-site cytopathologist, the experience of the institution's pathology staff, the imaging equipment available, and the skill of the operating physician. The pooled mean success rate for thoracic/pulmonary and non-musculoskeletal biopsies is 89% with a range of 77–96% for former and 70–90% for latter [18]. The technical success of our consultant interventional radiologists (Drs AG and BW), who will be doing most of the biopsies in this study with transthoracic biopsies, is at 95.9% over last 3 years (165 of the 172 biopsies gave a diagnostic yield).

Complications associated with PNB are subject to multiple factors such as the nature of the lesion, complexity of biopsy and technique of the operator. Hence it is always preferable for an operator to quote his/her own personal complication rate alongside the internationally accepted rates. Table 1 shows the personal complication rate for Drs AG and BW alongside the reported rates in

literature and the SIR-ACR (Society of Interventional Radiology- American College of Radiology) acceptable thresholds of complication rate for PNB.

Table 1. Complication rates following radiologically guided biopsies.

	Reported rates in literature (%)	SIR-ACR biopsy guidelines acceptable threshold (%)	Personal statistics for Dr AG over last 3 years	Personal statistics for Dr BW over last 12mths
**Thoracic Biopsy**				
Pneumothorax	12–45	45	16.8% (29/172)	32.2% (10/31)
Pneumothorax needing chest drain	2–15	20	4.6% (8/172)	3.2% (1/31)
Hemorrhagic complications	0.5	2	0.7 (1/172)	0 (0/31)
**Major complications with non-thoracic biopsies**				Total US guided biopsies previous 12 months (n = 104)
Liver	0.3–3.3	5	1% (1/95)	0
Renal	0.5–2.8	5	0 (0/20)	0
Other (retroperitoneum, paraspinal masses, peritoneal/pelvic masses)	0.1–3	6	0 (0/62)	0

6. Study Monitoring and Data Collection

6.1. Data collection

Case Record Forms (CRFs)

All data obtained in the study described in this protocol will be recorded on CRFs. The CRF for each subject will be presented in a folder. The CRF will be completed chronologically and updated regularly in order to reflect the most recent data on the patient included in the study.

Errors must be corrected by drawing a single line through the incorrect entry and by writing the new value as close as possible to the original. The correction must then be initialed and dated by an authorized person.

The investigator will add to the subject trial file, after completion of the study, any relevant post-trial information brought to his attention.

6.2. Data entry

A data manager will enter data into an electronic database in a password protected, user-designated computer in the office of the Department of Haematology-Oncology.

6.3. Maintenance of patients records

Clinical report forms (CRF) will be used to record data for this study. A copy of the CRF will be kept in the Department of Haematology-Oncology Office. All records will be kept for a period of 6 years following the date of study closure according to Singapore GCP guidelines.

7. Sample Size and Statistical Considerations

A total of 35 patients will be enrolled in this study. An additional twenty patients will be recruited in addition to the current number of patients already enrolled (15 patients) to allow for further safety analysis and analysis of clonal evolution of disease from diagnosis to subsequent

recurrence/s. This is a pilot feasibility study and no sample size calculation has been performed. The recruitment target has been established based on the cost of doing whole exome sequencing on 3 -4 CNBs/FNBs and germline matched DNA on each patient and services offered in kind by our collaborators at Singapore Immunology Network (Subhra Biswas) allowing for the increased total number of recruited patients to increase to a total of 35.

8. Informed Consent, Ethical Review and Regulatory Considerations

8.1. Informed Consent

The informed consent document will be used to explain the risks and benefits of study participation to the patient in layman terms before the patient is entered into the study.

The investigator is responsible to see that informed consent is obtained from each patient or legal representative and to obtain the appropriate signatures and dates on the informed consent document prior to the performance of any protocol procedures.

As used in this protocol, the term "informed consent" includes all consent and/or assent given by patients and their legal representatives.

Patient information

The responsible physician will inform the patient about the background of the study. The patient will be told of his or her right to withdraw from the study at any time without any penalty with regards to the continuation of care at this institution and by the same physicians as he chooses. The patient will be told that tissue samples obtained will be assigned unique patient numbers (UPN) to ensure patient confidentiality.

8.2. Ethical Review

Approval of the protocol and the informed consent document will be obtained from the institution's ethical review board before the study may begin.

The investigator will supply the following to the study site's ethical review board(s):

- The study protocol
- Informed consent document
- Relevant curricula vitae

8.3. Regulatory Considerations

This study will be conducted in accordance with the ethical principles stated in the most recent version of the Singapore guidelines on good clinical practice (GCP).

9. Publication Policy

Authors shall be those who have made a significant contribution to the overall success of the study. This contribution will be assessed, in part but not entirely, in terms of cases contributed, generation of data and analysis of results, and will be reviewed by the principal investigator. Materials shall not be submitted for presentation or publication without review and approval of the principal investigator.

References

1. Hanahan, D., Weinberg, R.A. The hallmarks of cancer. *Cell* **2000,** *100*, 57–70.
2. Greenman, C.; Stephens, P.; Smith R.; Dalgliesh, G.L.; Hunter, C.; Bignell, G.; Davies, H.; Teague, J.; Butler, A.; Stevens, C.; et al. Patterns of somatic mutation in human cancer genomes. *Nature* **2007**, *446*, 153–158.
3. MacConaill, L.E.; Campbell, C.D.; Kehoe, S.M.; Bass, A.J.; Hatton, C.; Niu, L.; Davis, M.; Yao, K.; Hanna, M.; Mondal, C.; et al. Profiling critical cancer gene mutations in clinical tumor samples. *Plos One* **2009**, *4*, e7887.
4. Dias-Santagata, D.; Akhavanfard, S.; David, S.S.; Vernovsky, K.; Kuhlmann, G.; Boisvert, S.L.; Stubbs, H.;

McDermott, U.; Settleman, J.; Kwak, E.L.; et al. Rapid targeted mutational analysis of human tumours: A clinical platform to guide personalized cancer medicine. *Embo Mol. Med.* **2010**, *2*, 146–158.
5. Garber, K. Trial offers early test case for personalized medicine. *J. Natl. Cancer Inst.* **2009**, *101*, 136–138.
6. Von Hoff, D.D.; LoRusso, P.M.; Rudin, C.M.; Reddy, J.C.; Yauch, R.L.; Tibes, R.; Weiss, G.J.; Borad, M.J.; Hann, C.L.; Brahmer, J.R.; et al. Inhibition of the hedgehog pathway in advanced basal-cell carcinoma. *N. Engl. J. Med.* **2009**, *361*, 1164–1172.
7. Fong, P.C.; Yap, T.A.; Boss, D.S.; Carden, C.P.; Mergui-Roelvink, M.; Gourley, C.; De Greve, J.; Lubinski, J.; Shanley, S.; Messiou, C.; et al. Poly(ADP)-ribose polymerase inhibition: frequent durable responses in BRCA carrier ovarian cancer correlating with platinum free interval. *J. Clin. Oncol.* **2010**, *28*, 2512–2519.
8. Flaherty, K.; Puzanov, I.; Sosman, J.; Kim, K.; Ribas, A.; McArthurR, J.; Grippo, J.F.; Nolo, K.; Chapman, P.; et al. Phase I study of PLX4032: Proof of concept for V600E BRAF mutation as a therapeutic target in human cancer. *J. Clin. Oncol.* **2009**, *27*, 9000.
9. Kwak, E.L.; Camidge, D.R.; Clark, J.; Shapiro, J.I.; Maki, R.G.; RatainB. Solomon, M.J.; Bang, Y.; Ou, S.; Salgia, R.; et al. Clinical activity observed in a phase I dose escalation trial of an oral c-met and ALK inhibitor, PF-02341066. *J. Clin. Oncol.* **2009**, *27*, 3509.
10. Heppner, G.H.; Miller, B.E. Tumor heterogeneity: Biological implications and therapeutic consequences. *Cancer Metastasis Rev.* **1983**, *2*, 5–23.
11. Iwasa, Y.; Michor, F. Evolutionary Dynamics of Intratumor Heterogeneity. *Plos One* **2011**, *6*, e17866.
12. Diaz-Cano, S.J. Tumor Heterogeneity: Mechanisms and Bases for a Reliable Application of Molecular Marker Design. *Int. J. Mol. Sci.* **2012**, *13*, 1951–2011.
13. Diaz-Cano, S.J. Designing a molecular analysis of clonality in tumours. *J. Pathol.* **2000**, *191*, 343–344.
14. Shah, S.P.; Morin, R.D.; Khattra, J.; Prentice, L.; Pugh, T.; Burleigh, A.; Delaney, A.; Gelmon, K.; Guliany, R.; Senz, J.; et al. Mutational evolution in a lobular breast tumour profiled at single nucleotide resolution. *Nature* **2009**, *461*, 809–813.
15. Klein, C.A.; Schmidt-Kittler, O.; Schardt, J.A.; Pantel, K.; Speicher, M.R.; Riethmüller, G. Comparative genomic hybridization, loss of heterozygosity, and DNA sequence analysis of single cells. *Proc. Natl. Acad. Sci.* **1999**, *96*, 4494.
16. González-García, I.; Solé, R.V.; Costa, J. Metapopulation dynamics and spatial heterogeneity in cancers. *Proc. Natl. Acad. Sci.* **2002**, *99*, 13085.
17. Wistuba, I.I.; Gelovani, J.G.; Jacoby, J.J.; Davis, S.E.; Herbst, R.S. Methodological and practical challenges for personalized cancer therapies. *Nat. Rev. Clin. Oncol.* **2011,** *8*, 135–141.
18. Gupta, S.; Wallace, M.J.; Cardella, J.F.; Kundu, S.; Miller, D.L.; Rose, S.C.; Society of Interventional Radiology Standards of Practice Committee. Quality improvement guidelines for percutaneous needle biopsy. *J. Vasc. Interv. Radiol.* **2010,** *21*, 969–975.
